# Long-Term Transcriptomic Changes and Cardiomyocyte Hyperpolyploidy after Lactose Intolerance in Neonatal Rats

**DOI:** 10.3390/ijms24087063

**Published:** 2023-04-11

**Authors:** Olga V. Anatskaya, Andrey L. Runov, Sergey V. Ponomartsev, Maxim S. Vonsky, Artem U. Elmuratov, Alexander E. Vinogradov

**Affiliations:** 1 Institute of Cytology RAS, Saint Petersburg 194064, Russia; 2 The D.I. Mendeleev All-Russian Institute for Metrology (VNIIM), Moskovsky ave 19, Saint Petersburg 190005, Russia; 3 Almazov Medical Research Centre, Akkuratova Street 2, Saint Petersburg 197341, Russia; 4 Medical Genetics Centre Genotek, Nastavnichesky Alley 17-1-15, Moscow 105120, Russia

**Keywords:** cardiomyocyte, polyploidy, qualitative transcriptome analysis, developmental programming of adult heart diseases, neonatal lactose intolerance, DNA instability, inflammation, fibrosis, thyroid deficiency, glutathione deficiency

## Abstract

Many cardiovascular diseases originate from growth retardation, inflammation, and malnutrition during early postnatal development. The nature of this phenomenon is not completely understood. Here we aimed to verify the hypothesis that systemic inflammation triggered by neonatal lactose intolerance (NLI) may exert long-term pathologic effects on cardiac developmental programs and cardiomyocyte transcriptome regulation. Using the rat model of NLI triggered by lactase overloading with lactose and the methods of cytophotometry, image analysis, and mRNA-seq, we evaluated cardiomyocyte ploidy, signs of DNA damage, and NLI-associated long-term transcriptomic changes of genes and gene modules that differed qualitatively (i.e., were switched on or switched off) in the experiment vs. the control. Our data indicated that NLI triggers the long-term animal growth retardation, cardiomyocyte hyperpolyploidy, and extensive transcriptomic rearrangements. Many of these rearrangements are known as manifestations of heart pathologies, including DNA and telomere instability, inflammation, fibrosis, and reactivation of fetal gene program. Moreover, bioinformatic analysis identified possible causes of these pathologic traits, including the impaired signaling via thyroid hormone, calcium, and glutathione. We also found transcriptomic manifestations of increased cardiomyocyte polyploidy, such as the induction of gene modules related to open chromatin, e.g., “negative regulation of chromosome organization”, “transcription” and “ribosome biogenesis”. These findings suggest that ploidy-related epigenetic alterations acquired in the neonatal period permanently rewire gene regulatory networks and alter cardiomyocyte transcriptome. Here we provided first evidence indicating that NLI can be an important trigger of developmental programming of adult cardiovascular disease. The obtained results can help to develop preventive strategies for reducing the NLI-associated adverse effects of inflammation on the developing cardiovascular system.

## 1. Introduction

A recent global survey has marked cardiovascular diseases as the leading causes of death in over hundred countries (GBD 2016 Causes of Death Collaborators 2017) [1]. Previously, a comprehensive study has shown that about half of all deaths in the USA were preventable [2]. Heart diseases are at the top of this list. Retrospective epidemiological data and long-term experimental studies indicate that many cardiovascular diseases (CVD), including hypertension, coronary heart disease, atherosclerosis, and cardiomyopathy may originate from the stressful condition during prenatal and early postnatal development [3,4]. Adverse growth conditions may irreversibly decrease cardiomyocyte number, alter cardiac microstructure, and change heart anatomy, thereby limiting organ functional capacity and increasing the risk of cardiovascular diseases later in life [5,6].

Much evidence confirms the developmental origin hypothesis for intrauterine environments. For example, the intrauterine growth retardation, maternal obesity, alcohol consumption, glucocorticoid treatment, hypoxia, and vitamin D deficiency increase the risk of hypertension, coronary heart disease, myocardial insulin resistance (reviewed in [4,7]). Recent studies indicate that developmental conditions can program health and disease via epigenetic mechanisms [8,9]. The relevance of the developmental origin hypothesis to the early postnatal period has been less investigated. At the same time, recent studies provided evidence that the disturbing of perinatal, neonatal, and early postnatal development may also outcome in adult diseases [10]. Thus, neonatal malnutrition, inflammation, and growth retardation are associated with a lifelong decrease in cardiomyocyte number, systolic and diastolic dysfunction, ischemia sensitivity, and high blood pressure [11,12,13,14]. In light of these observations, the searching for new early postnatal triggers of adult cardiovascular diseases acquires particular relevance and importance.

One of the fruitful approaches may be a comprehensive analysis of epidemiologic, evolutionary, and cross-species studies [8,15,16,17]. Therefore, we compared the data of epidemiological studies concerning the diseases triggering the main causes of developmental programming of CVD, including growth retardation, inflammation, and malnutrition in babies, toddlers, and children [18,19,20,21], and the literature describing the data on developmental adaptation of cardiovascular system in more than 30 bird and 40 mammal species [14,22,23,24,25,26]. The data of epidemiological studies revealed early life gastroenteritis of various etiologies [18,20,27,28,29,30]. Comparative cross-species analysis indicated that early life cardiac functional load can be an important trigger of the developmental programming of adult CVD, because it is inversely related to heart aerobic capacity in adults [23,24,25].

Early life gastroenteritis may increase heart functional load via tachyarrhythmia, cardiac palpitations, electrolytic and metabolic imbalance, and inflammatory cytokine over-production [20,30,31,32,33]. Moreover, even premature weaning can cause intestinal malabsorption via disturbance of intestinal villous length and crypt depth [34]. In accordance, our recent studies indicated that cryptosporidial gastroenteritis in neonatal rats in long-term cardiomyocyte increased polyploidy, remodeling, and overexpression of fetal isoform of myosin heavy chain (MYH7) [14,35,36]. Altogether, these adverse effects should inevitably alter normal trajectory of cardiac development and impair heart function. 

A common symptom of gastroenteritis of various etiologies challenged, for example, by dysbiosis, parasitosis, bacterial or viral infection, inflammation, or allergy is lactose intolerance originating from villous atrophy and lactase deficiency [37,38,39]. Medical statistics indicates that dairy products and milk intake may increase risk of ischemic heart disease [40,41]. The results of the experimental studies found that excessive lactose in diet decreases cardiac contractility and induces heart atrophy and arrhythmia [42,43]. Accordingly, the data obtained with galactose, which is a product of lactose decomposition by intestinal lactase, indicate that galactose accelerates heart aging [44], oxidative damage [32], decreases telomerase activity, and increases markers of senescence [44,45]. Thus, the experimental studies indicate that both decomposed and undigested forms of lactose exert negative effects on cardiac structure and function. 

Currently, nothing is known about the long-term effects of neonatal lactose intolerance (NLI) on cardiac structure and function. The presented study aimed to verify the hypothesis that NLI may exert long-term detrimental effects on cardiac developmental programs via cardiac dysfunction, cardiomyocyte remodeling, and manifestations of stress response, including increased cardiomyocyte polyploidy and myofibril fetalization.

Reactivation of fetal gene program and increased cardiomyocyte ploidy were well documented for numerous heart diseases, including ischemic diseases, hypertension, congenital heart diseases, and cardiomyopathies [46,47,48,49,50,51]. Here we provide first evidence that early postnatal lactose intolerance challenged by lactase overloading results in the long-term cardiomyocyte hyperpolyploidization, DNA instability, and extensive transcriptomic changes that are features of heart disease and hyperpolyploidy.

We concentrated here on the most strongly deregulated genes, with a discrete difference between the experiment and the control (switched on/off genes). Such qualitative differences are a sign that the genome is undergoing substantial changes in expression possibly related to chromatin remodeling [52,53,54]. The switched on/off genes were revealed previously both on the transcriptome and proteome levels and are more typical for development and cancer cells, which experience profound changes [53,54,55,56]. Here we found the on/off gene switching in the NLI response, suggesting that it involves deep, qualitative transcriptomic changes.

## 2. Results

### 2.1. Animals

**Figure 1:**
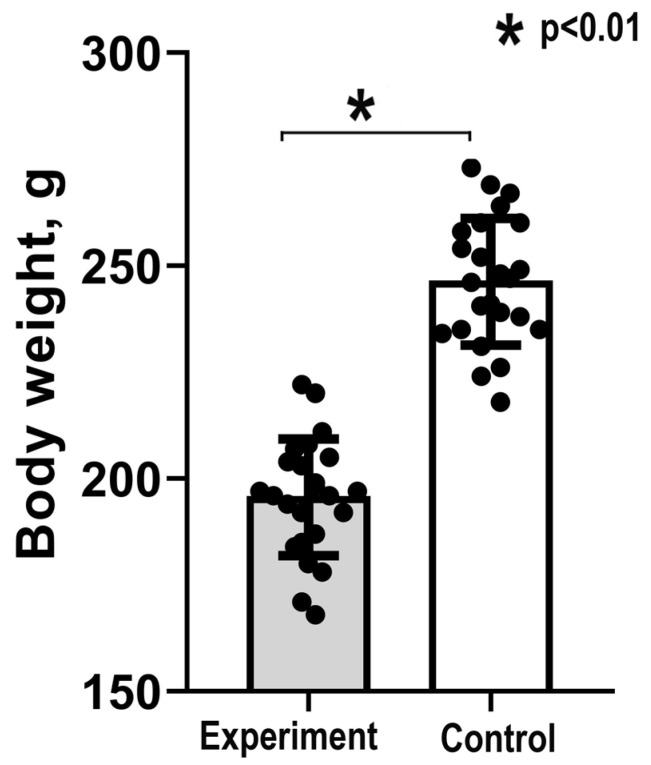
Body weight in the experimental and control animals. The figure illustrates the decreased body weight in the experimental animals compared with the control even after 4 months of recovery from NLI (Mann–Whitney and *t*-test, *p* < 0.01). The bars represent mean values; the error bars show confidence intervals (CI), *p* = 0.95; the points represent separate values.

At the age of 140 days, i.e., at four months after lactose treatment, the experimental animals were smaller than the control ones. The results of weighting indicated that the control vs. the experiment difference in body mass comprised about 25% (248 ± 9.2 g in the control vs. 199 ± 7.3 g in the experiment, Mann–Whitney, *p* < 0.01 for the experiment vs. the control difference; Figure 1). This result indicates that NLI is associated with animal growth retardation suggesting that the treatment causes the long-term physiological rearrangements.

### 2.2. Cardiomyocyte DNA Instability and Polyploidy

**Figure 2:**
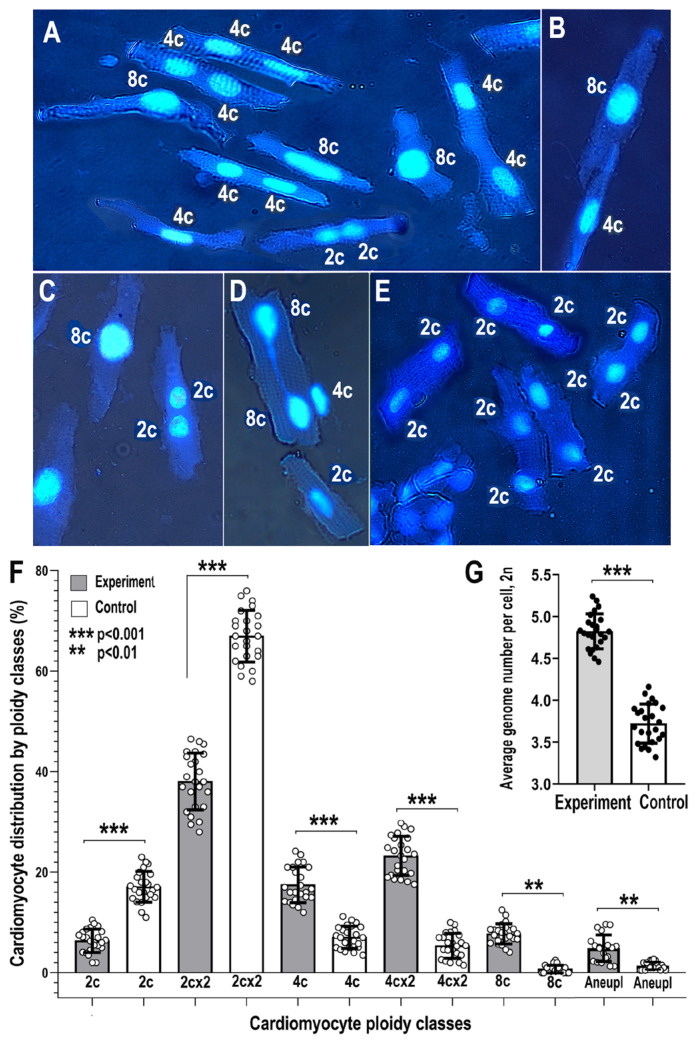
Increased cardiomyocyte ploidy and genetic instability in the experimental animals compared to the control. (**A**–**C**)—cardiomyocytes with polyploid nuclei containing 4 or 8 genomes from the NLI survived rats. (**D**)—cardiomyocytes of the experimental animal with two octaploid nuclei with a bridge between them pointing to DNA instability. This image illustrates DNA instability after NLI. (**E**)—cardiomyocytes of the control animals with diploid nuclei. This image demonstrates the lower cardiomyocyte ploidy of the control animals compared to the experiment. Nuclei are stained with Hoechst 33258, double lightning, transmitted light and luminescence, phase contrast. Total magnification is ×200. (**F**)—distribution of cardiomyocytes by ploidy classes in the control and experimental animals. Figure illustrates the increased percentages of tetraploid, octaploid, and aneuploid cells in the experiment compared to the control. (**G**)—average cardiomyocyte ploidy levels. The figure illustrates the increase in the average cardiomyocyte genome number per cell in the experiment compared to the control. The values are presented as mean ± confidence intervals (CI), *p* = 0.95. The statistical significance of differences is indicated in the Figure (Mann–Whitney).

Cytophotometry of DAPI-stained nuclei of the isolated left ventricle (LV) cardiomyocytes revealed increased cell ploidy in the experiment vs. the control. Genome accumulation occurred mainly due to the increased percent of tetraploid cells with two diploid nuclei or one tetraploid nucleus and due to the accumulation of octaploid cells with one octoploid nucleus or two tetraploid nuclei. Figure 2 illustrates the cardiomyocytes of experimental animals with tetra- and octoploid nuclei (A,D) and cardiomyocytes of the control animals with diploid nuclei (E). Figure 2D,F shows cardiomyocyte distribution by ploidy classes and average cardiomyocyte ploidy in the experimental and control animals. It can be seen that NLI induces a statistically significant increase in the percentage of 2cx2, 4c, 4cx2, and 8c cardiomyocytes, and increases the average genome number per cell (i.e., average cell ploidy) by about 20% (4.7 ± 0.27 c in the experiment vs. 3.7 ± 0.23 c in the control, Mann–Whitney, *p* < 0.005 for the experiment vs. the control difference). This result indicates that early neonatal lactose treatment triggers long-term cardiomyocyte hyperpolyploidization in rats.

Unexpectedly, besides the increased polyploidy, the cardiomyocytes of the experimental animals revealed the chromatin bridges between the nuclei (Figure 2D). Bridges between nuclei is a feature of genetic instability [57,58]. In the experimental animals, the percentage of cells with bridges comprised 1.8 ± 0.4%. In the control animals, we did not find cells with bridges (1.8 ± 1.6% vs. 0.0, Mann–Whitney, *p* < 0.001). The other feature of genetic instability is the increased percentage of cells with aneuploid nuclei [59]. Figure 2F shows the increased percent of aneuploid cells in experiment vs. the control (5.1 ± 1.6% vs. 0.94 ± 0.05%, Mann–Whitney, *p* < 0.004).

### 2.3. Cardiomyocyte Protein Content Evaluation

Quantitative image analysis indicated that NLI is associated with the long-term cardiomyocyte atrophy evident from the decrease in the protein content of isolated cardiomyocyte in the experiment vs. the control. Supplementary Figure S1A,B illustrate naphthol yellow stained cardiomyocytes from the NLI survived (A) and the control animals (B). Supplementary Figure S1C,D demonstrate the bar charts and individual points for the data related to the protein content per cardiomyocyte (C) and per one genome of cardiomyocyte (D). It is clearly seen that NLI decreases the protein amount per cardiomyocyte by about 1.28 folds (Mann–Whitney, *p* < 0.03 for the experiment vs. the control difference) thereby increasing the DNA/protein ratio in a cell. This effect is further enhanced by polyploidization. As a result, the protein amount per genome decreases almost by 1.7 folds (Mann–Whitney, *p* < 0.01 for the experiment vs. the control difference).

### 2.4. Cardiac Left Ventricle (LV) Transcriptome Changes in Adult Rats after NLI

#### 2.4.1. General Picture

In our model, NLI induced animal growth retardation, long-term cardiomyocyte hyperpolyploidization, and features of DNA instability. To investigate the molecular nature of these pathological changes and to find the most prominent NLI-induced transcriptomic features, we performed RNA-seq of mRNA extracted from the LV apical part of 140 days old animals that survived NLI. Overall, RNA-seq identified 13,742 genes. To reveal the most strongly differentially expressed genes (DEGs), we selected those genes that demonstrated the qualitative difference in expression, i.e., were expressed in the experiment and were not expressed in the control (i.e., switched on in the experiment) or, on the contrary, were expressed in the control and were not expressed in the experiment (i.e., switched off in the experiment). These genes are also termed as toggle genes [56]. The identification of the switched off and switched on genes enables the identification of NLI-associated qualitative changes in gene regulatory networks and protein interaction networks for proteins encoded by these genes.

**Figure 3:**
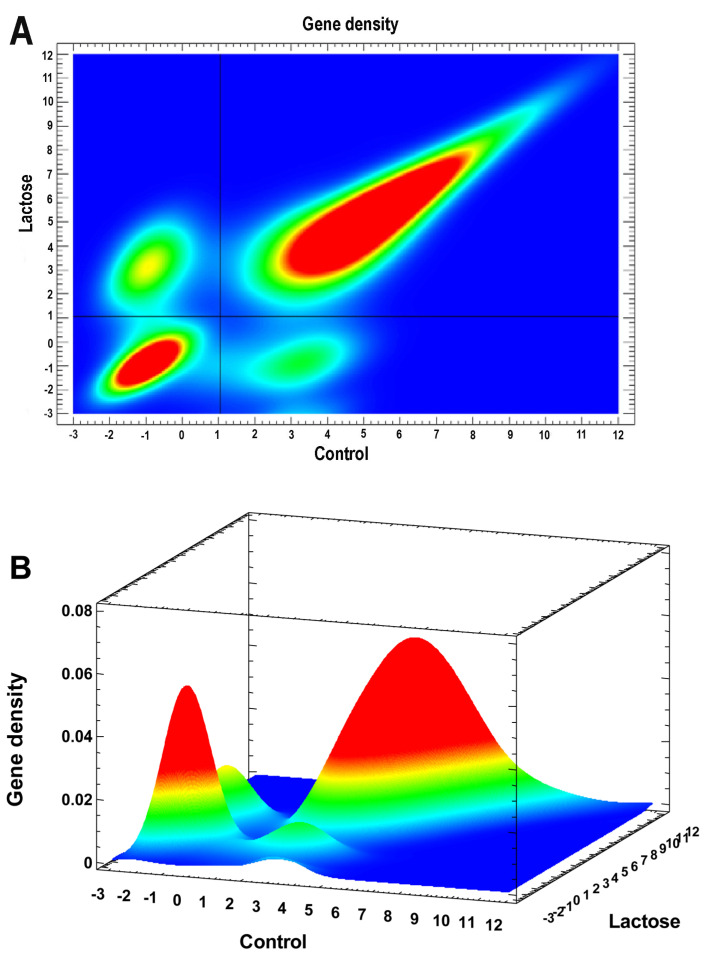
Two dimensional histograms of gene expression levels in the experiment (NLI) vs. the control. (**A**)—2D representation. The four peaks of gene expression density are visible: the lower left (no expression both in experiment and the control), the upper left (expression only in the experiment, i.e., switched on), the lower right (expression only in the control, i.e., switched off), and the upper right (expression both in experiment and the control). The cutoff values are shown by the horizontal and vertical black lines. The genes from the upper left (switched on) and the lower right (switched off) rectangles were analyzed. The significance of pairwise differences between any peaks are *p* < 10^−24^ at least (both Mann–Whitney and *t*-test). (**B**)—3D representation (the color scale of gene density is seen).

The switched on/off (toggle) genes were revealed similarly with the procedure that was used in [56], but we enhanced it using a two-dimensional histogram instead of a simple scatter plot, not showing gene densities when gene points become too dense and overlapping. Therefore, a two-dimensional histogram allows better discrimination of gaps between peaks, thereby avoiding arbitrary cutoffs. The problem with determining whether the genes are switched on/off or just gradually changing expression is that many genes can show expression not at zero level, but very close to it (especially after normalization). However, it is not clear whether this expression is functional or just a transcriptional (or technical) noise [60,61,62]. Therefore, we considered the peaks of gene density on the two-dimensional histogram as the switched on/off criterion (Figure 3A,B). The gaps between the peaks show the qualitative (discrete) changes in expression corresponding to transitions between different states, i.e., switching the gene on or off. The significance of pairwise differences between any peaks is *p* < 10^−24^ at least (both in Mann–Whitney and *t*-test). This selection gave 1322 “on” and 1028 “off” genes.

To identify the biological pathways enriching DEGs with maximum stringency, we applied the further multistep purification of signal from noise for switched on/off genes, which was based on the protein interaction network analysis. This was performed because we wanted to exclude possible spurious hits and to reveal the strongest effects in the experiment vs. the control. First, using the String server [63], we obtained protein–protein interactions of high confidence for switched on/off DEGs. This step gave 778 switched on and 552 switched off DEGs. Then, to reveal the most important genes (hubs), we selected nodes with degree above 2 (i.e., nodes with >2 interactions). This step provided 496 upregulated and 355 downregulated genes. Then, to identify the tight functional gene clusters among these genes, we applied the “Glay clustering” algorithm implemented in the Cytoscape [64] (version 3.9.1) as previously [65]. This is a method of choice for our purpose because the Glay algorithm provides a complete clustering of large interaction networks [65,66,67].

**Figure 4:**
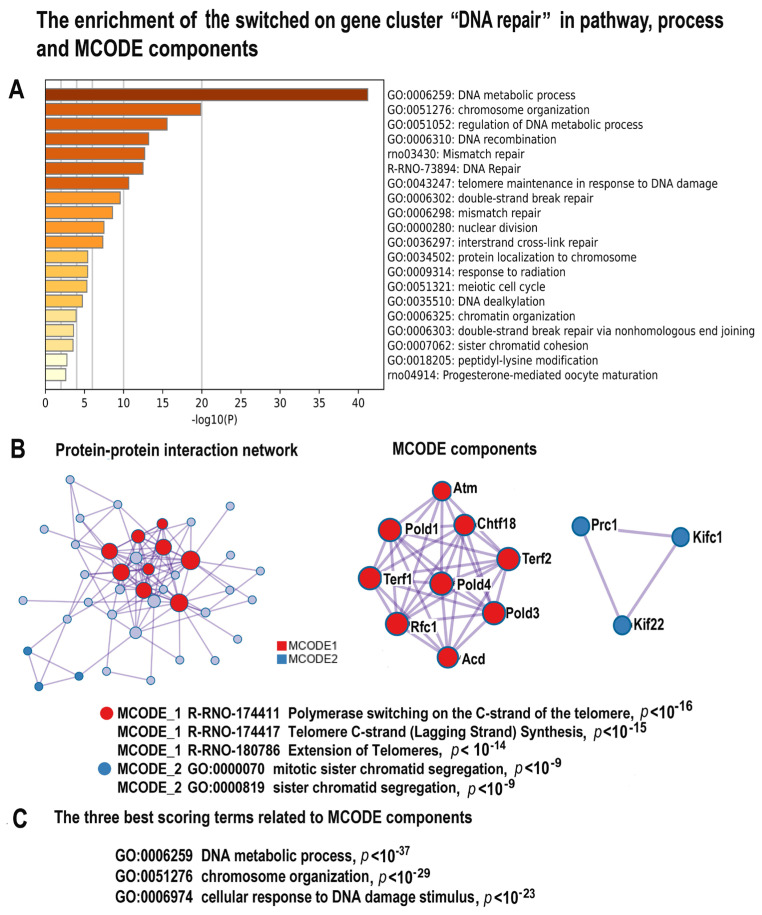
The enrichment of NLI-induced genes related to the cluster “DNA Repair” in gene pathways and in processes and molecular complexes. (**A**)—Bar graph of enriched terms related to gene modules and processes across the gene cluster related to DNA repair. The statistical significance of enrichment is shown at the *X*-axis (−log10 ((*p*))). (**B**)—Protein–protein interaction network and MCODE components (or densely connected network components) that were identified in the gene list. The network and MCODE components were constructed on the base of physical interactions taken from String server (physical score > 0.4). The coding by color squares reflects the MCODE components. The coding by color circles indicates the results of MCODE component pathway and process enrichment analysis. (**C**)—The three best-scoring terms related to MCODE components.

**Figure 5:**
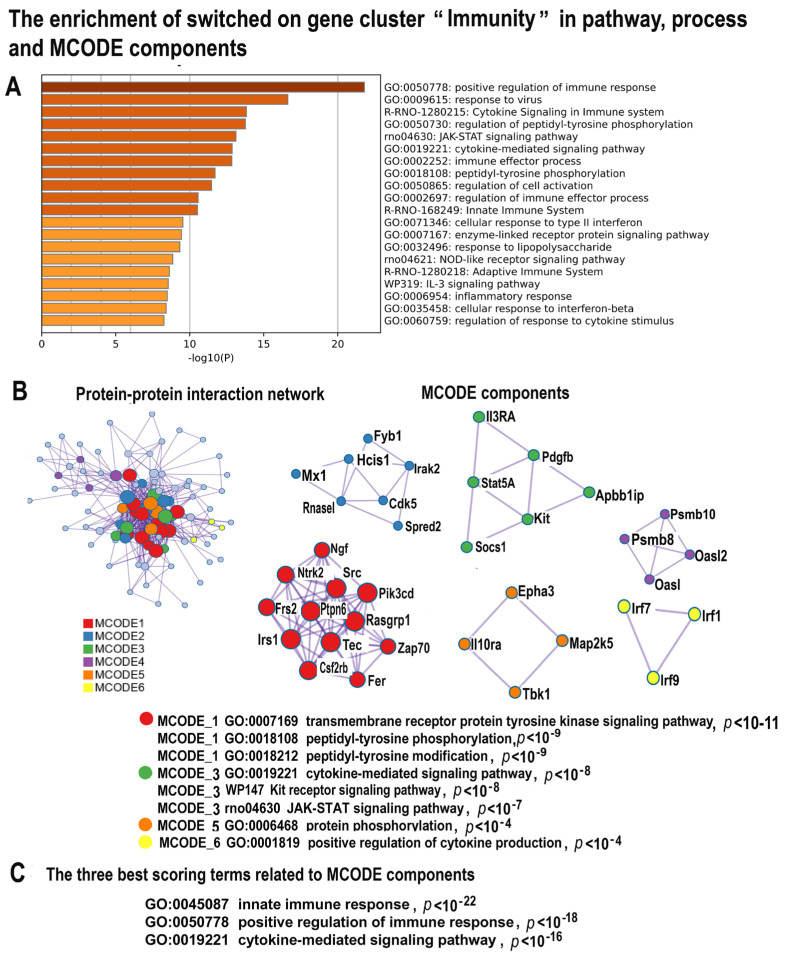
The enrichment of NLI-induced genes related to the cluster “Immunity” in gene pathways, processes, and molecular complexes. (**A**)—Bar graph of enriched terms related to gene pathways and processes across the gene cluster related to immunity. The statistical significance of enrichment is shown in *X*-axis (−log10(*p*)). (**B**)—Protein–protein interaction network and MCODE complexes that were identified in the gene list. The network and MCODE components were constructed on the base of physical interactions taken from the String server (physical score > 0.4). The coding by color square reflects the MCODE components. The coding by color circles indicates the results of MCODE component pathway and process enrichment analysis. (**C**)—The three best-scoring terms related to MCODE.

**Figure 6:**
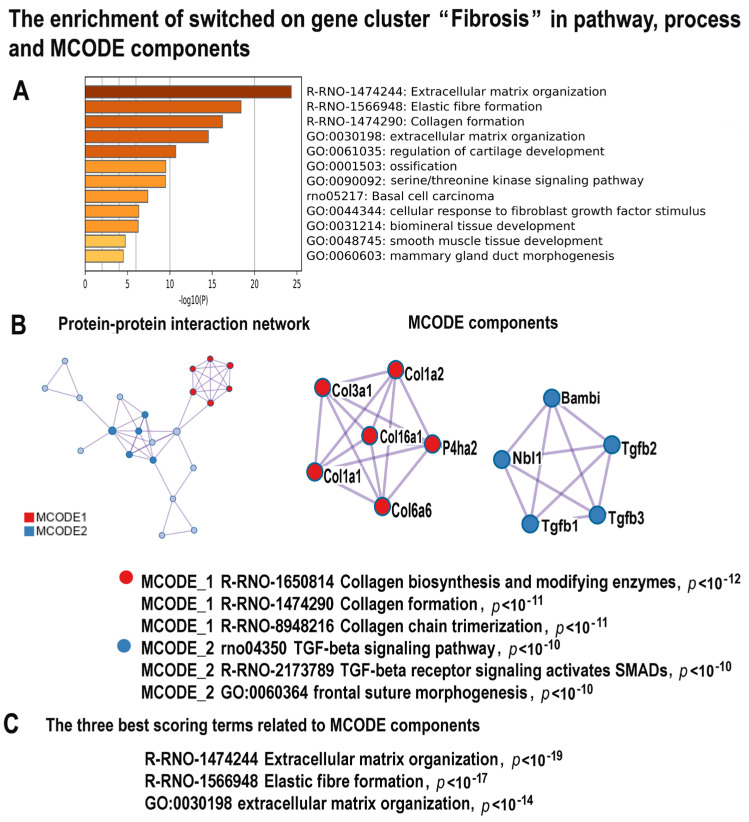
The enrichment of NLI−induced genes related to the cluster “Fibrosis” in gene pathway and process and in molecular complexes. (**A**)—Bar graph of enriched terms related to gene pathways and processes across the gene cluster related to fibrosis. The statistical significance of enrichment is shown in *X*-axis (−log10(*p*)). (**B**)—Protein–protein interaction network and MCODE components (or densely connected network components) were identified in the gene list. The network and MCODE components were constructed on the base of physical interactions taken from the String server (physical score > 0.4). The coding by color square reflects the MCODE components. The coding by color circles indicates the results of MCODE component pathway and process enrichment analysis. (**C**)—The three best-scoring terms related to MCODE components.

**Figure 7:**
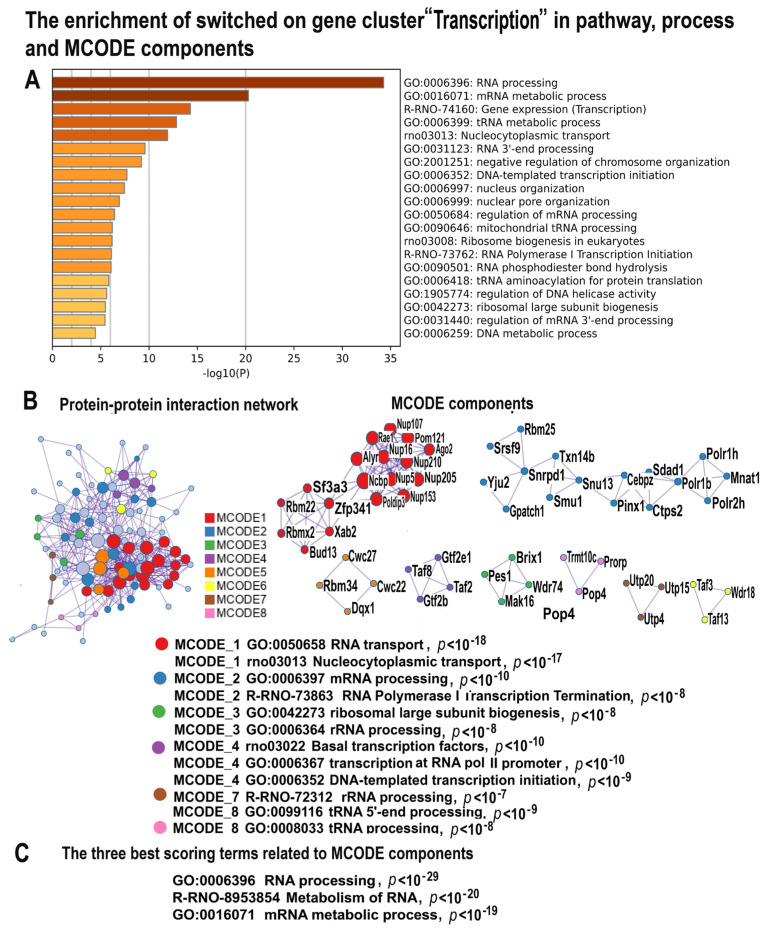
The enrichment of NLI−induced genes related to the cluster “Transcription” in gene pathway and process and in molecular complexes. (**A**)—Bar graph of enriched terms related to gene pathways and processes across the gene cluster related to transcription. The statistical significance of enrichment is shown in *X*-axis (−log10(*p*)). (**B**)—Protein–protein interaction network and MCODE components (or densely connected network components) were identified in the gene list. The network and MCODE components were constructed on the base of physical interactions taken from the String server (physical score > 0.4). The coding by color square reflects the MCODE components. The coding by color circles indicates the results of the MCODE component pathway and process enrichment analysis. (**C**)—The three best-scoring terms related to MCODE components.

**Figure 8:**
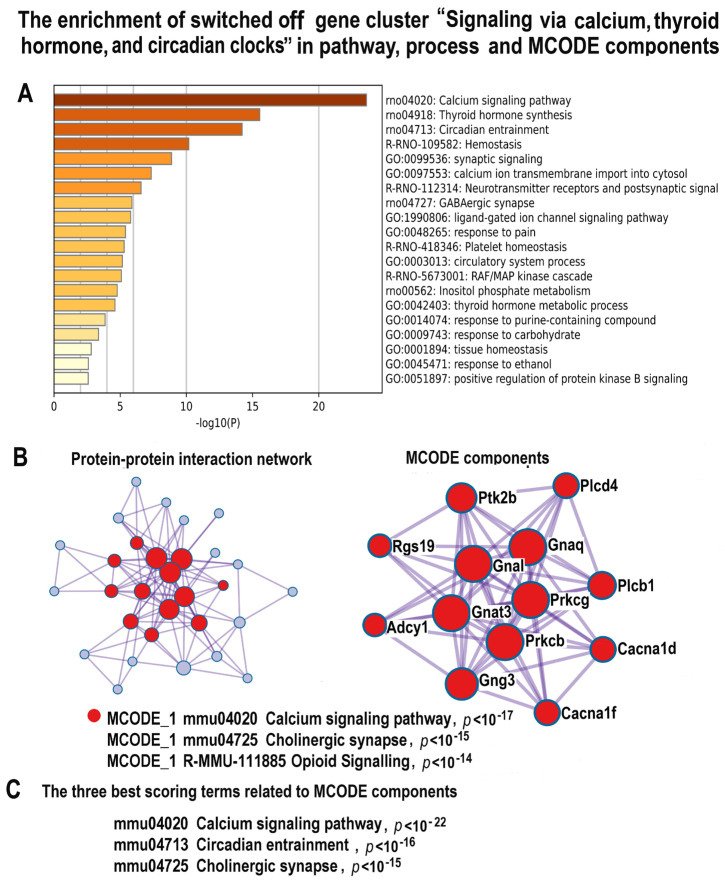
The enrichment of NLI−inhibited genes related to the cluster “Signaling via calcium, thyroid and circadian clocks” in gene pathway and process and in molecular complexes. (**A**)—Bar graph of enriched terms related to gene pathways and processes across the gene cluster related to signaling via calcium, thyroid hormone, and circadian clocks. The statistical significance of enrichment is shown in *X*-axis (−log10(*p*)). (**B**)—Protein–protein interaction network and MCODE components (or densely connected network components) were identified in the gene list. The network and MCODE components were constructed on the base of physical interactions taken from the String server (physical score > 0.4). The coding by color square reflects the MCODE components. The coding by color circles indicates the results of the MCODE component pathway and process enrichment analysis. (**C**)—The three best-scoring terms related to MCODE components.

**Figure 9:**
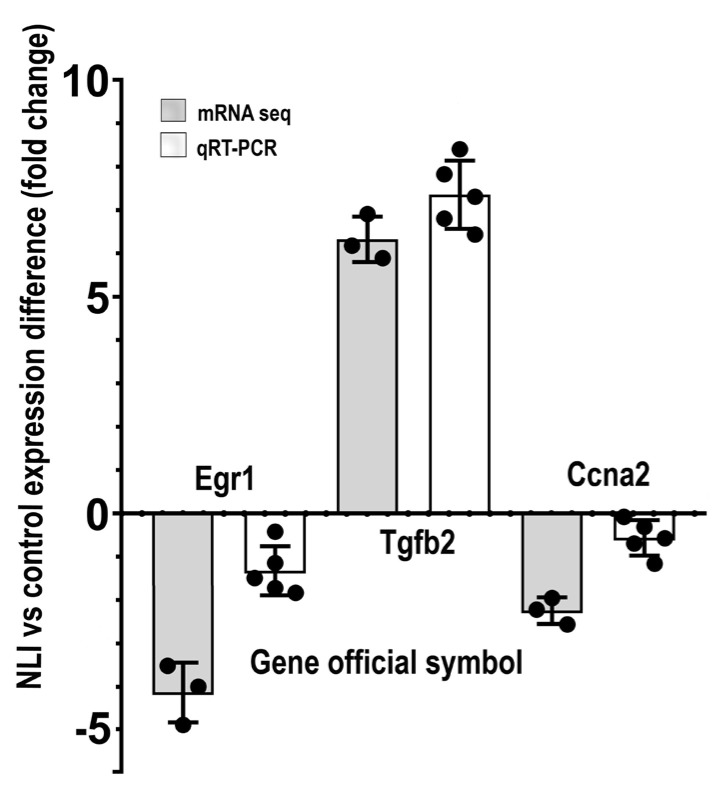
Validation of mRNA−seq data by qRT−PCR for Egr1, Tgfb2 and Ccna2. The bars represent mean values; the error bars show confidence intervals (CI), *p* = 0.95; the points represent separate values. The figure illustrates concordance between the data on the experiment vs. the control gene expression difference obtained with the mRNA−seq and qRT−PCR. The significance level for differences is *p* < 0.05 at least (Mann−Whitney and *t*−test).

**Figure 10:**
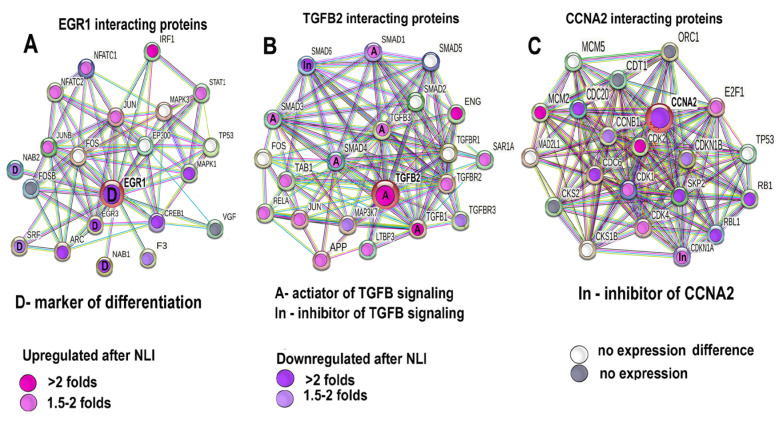
Protein interaction networks for proteins encoding for Egr1, Tgfb2, Ccna2, and their closest interactants matched with the data on the expression difference for the experiment vs. the control defined by mRNA–seq. (**A**)—Protein interaction networks for Egr1. It can be seen that EGR1 decreases expression together with its direct regulators and markers of differentiation Nab1, Nab2, Egr1, and Srf [105,106,107]. (**B**)—Protein interaction networks for TGFB2 indicates that Tgfb2 is upregulated together with the upregulation of its activators Tgfb1, 3 and Smad 3, 4, and the downregulation of its inhibitor Smad 6 [108]. (**C**)—Protein interaction network for Ccna2 demonstrates that Ccna2 is downregulated together with the upregulation of its inhibitor Cdkn1a [71,109]. The network is constructed for the 20 closest interactants of Egr1, Tgfb2, and Ccna2.

**Figure 11:**
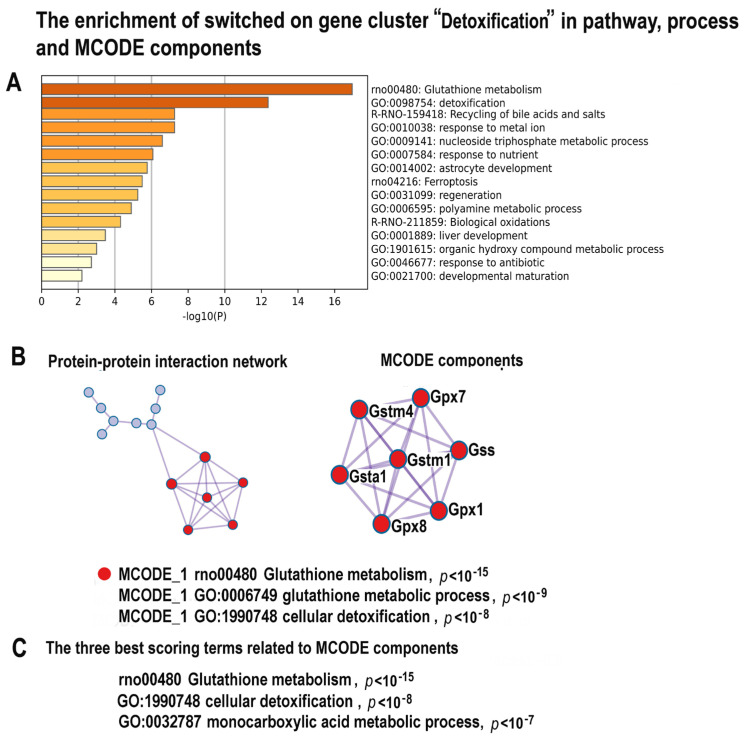
The enrichment of NLI−inhibited genes related to the cluster “Detoxication” in gene pathway and process and molecular complexes. (**A**)—Bar graph of enriched terms related to gene pathways and processes across the gene cluster related to detoxification, colored by different *p*-values. (**B**)—Protein–protein interaction network and MCODE components (or densely connected network components) were identified in the gene list. The network and MCODE components were constructed on the base of physical interactions taken from String server (physical score > 0.4). The coding by color square reflects the MCODE components. The coding by color circles indicates the results of MCODE component pathway and process enrichment analysis. (**C**)—The three best-scoring terms related to MCODE components.

Glay clustering revealed the four large tight clusters (containing above 10 genes) among the switched on genes. Specifically, the clusters are related to DNA instability, immunity, transcription, and fibrosis (unifying 369 genes). Among the switched off genes, there were the two such clusters. They are the cluster related to signaling via calcium, thyroid hormone, and circadian clocks, and the cluster related to glutathione signaling and detoxication (unifying 93 genes). (Supplementary Tables S1 and S2 present the lists of genes from the clusters of switched on and switched off genes). These clusters were analyzed with the Metascape server [68]. This server allows revealing biological features by means of term, pathway, and process enrichment analysis. Moreover, Metascape performs protein–protein interaction (PPI) enrichment analysis with physical interactions and constructs the network containing the subset of proteins that form physical interactions with at least one other member in the list. Metascape identifies molecular complexes via the Molecular Complex Detection (MCODE) algorithm [69]. Metascape analysis of Glay clusters revealed alterations in basic biological processes and molecular functions in the experiment vs. the control (Figure 4, Figure 5, Figure 6, Figure 7, Figure 8, Figure 9, Figure 10 and Figure 11; Supplementary Tables S1 and S2). Thus, we supported the analysis of switched on/off DEGs by the interactome analysis, revealing not just separate DEGs but DEGs forming the dense protein interaction clusters. This procedure excludes possible spurious hits which may be associated with separate genes. The higher robustness of analysis of functional gene groups compared with separate genes was discussed previously [70,71]. The individual gene changes not consolidated at the higher level (i.e., dispersed chaotically across functional gene groups) can be spurious [71,72].

Below we described the revealed findings.

#### 2.4.2. NLI Is Associated with Long-Term Persistence of Features of DNA Instability and DNA Damage Response

The most important feature associated with the long-term effects of NLI is DNA instability. Metascape analysis identified the cluster consisting of 55 upregulated genes enriched in several gene modules related to DNA repair, including “Mismatch repair”, “Base excision repair”, “Double strand break repair”, and “DNA recombination”, confirming the association between NLI and extensive DNA damage (Figure 4A–C; Supplementary Figure S2, Supplementary Tables S1 and S3). Our data also revealed the highly significant enrichment in gene modules related to telomeres, “Telomere maintenance in response to DNA damage” (Figure 4A) and “Telomere C-strand” and “Extension of telomeres” (Figure 4B). Moreover, the MCODE analysis identified the molecular complex related to the telomere (Figure 4B). This complex includes well-established markers of telomere maintenance Terf1 and Terf2 and the marker of DNA double strand break repair ATM, thus confirming that NLI induces telomere damage. In accordance, the three gene modules enriched for the switched on DEGs with the highest significance include a module related to DNA response to DNA damage stimulus (Figure 4C). In addition, the DNA repair-related cluster demonstrated enrichment in the pathways related to meiosis and progesterone-mediated oocyte maturation (Figure 4A). This result points to the manifestation of stemness and programs of female gametogenesis [73]. Thus, from our results, NLI is associated with the long-term DNA instability, telomere injure, and features of stemness. All these features were previously associated with cardiac diseases and failure [74,75].

#### 2.4.3. NLI Induces Gene Modules Related to Immunity and Inflammation

Metascape analysis indicated that the large cluster identified by Glay clustering (146 genes) includes the upregulated genes implicated in innate immunity, JAK-STAT signaling, response to virus, and interferon and cytokine production (Figure 5A–C; Supplementary Figure S3, Supplementary Tables S1 and S4). In accordance, the MCODE analysis revealed the molecular complex related to JAK-STAT and c-Kit signaling (Figure 5B). This complex includes the important regulators of immune response, such as Stat5A, c-Kit, and Il3RA (Figure 5A,B). The MCODE analysis also found the complex involved in positive regulation of cytokine production via Irf7, Irf1 and Irf9 (Figure 5B). The three gene modules that enrich the immunity-related cluster with the highest significance (*p* < 10^−16^) are presented by the “Cytokine mediated signaling pathway”, “Positive regulation of immune response”, and “Innate immune response” (Figure 5C). The induction of the JAK-STAT pathway and the pathways involved in cytokine and interferon production points to systemic inflammation generated by these tightly intertwined pathways associated with an increased risk of cardiovascular diseases, cardiomyopathy, and heart failure [76,77]. One more feature of cardiovascular pathology associated with this gene cluster is the manifestation of the fetal gene program. It is seen from the MCODE complexes and pathways related to JAK-STAT and c-Kit signaling (Figure 5C) that are involved in pluripotency maintenance [76,78,79,80,81,82,83].

#### 2.4.4. NLI Promotes Fibrosis

In accordance with the well-established association between cardiomyopathy, heart failure, and fibrosis [84], our data identified a tight cluster of 29 upregulated genes related to collagen biosynthesis and formation, elastic fiber formation, extracellular matrix, and ossification (Figure 6A–C, Supplementary Figure S4, Supplementary Tables S1 and S5). The MCODE analysis revealed the molecular complex involved in TGF beta signaling that includes the regulators of pro-fibrotic signaling Tgfb1, Tgfb2, Tgfb3, Nbl1, Bambi, and the molecular complex related to collagen biosynthesis and extracellular matrix production containing regulators Col3A, Col1a2, Col16a1, Col1a1, and Col6a6 (Figure 5B,C). It is well established that TGF beta signaling and collagen induce pro-fibrotic processes in multiple organs, including the heart [85], which provides additional evidence for the relationship between NLI and cardiac fibrosis.

#### 2.4.5. NLI Activates Gene Modules Related to Transcription and Ribosome Biogenesis

The largest Glay cluster (164 genes) includes genes related to transcription. Pathway and network enrichment analysis identified the high significance of enrichment for modules implicated in gene expression, transcription, and ribosome biogenesis (Figure 7A–C, Supplementary Figure S5, Supplementary Tables S1 and S6). This is evident from the terms “Gene expression, transcription”, “mRNA processing”, “DNA-templated transcription initiation”, “rRNA metabolism”, and “Ribosome biogenesis” enriching for cluster “Transcription” (Figure 6A–C). In accordance, the MCODE complex investigation found complexes implicated in RNA transport, mRNA and tRNA processing, basal transcription (regulated by Taf8, Taf2, Gtf2B and Gtf2E1), and ribosome biogenesis (regulated by Brix1, Pes1, Mak16). The activation of transcription and ribosome biogenesis may be the consequence of the transcriptionally permissive chromatin state originating from polyploidy, genetic instability, and other factors related to stress [79,86,87,88,89,90,91,92,93]. This suggestion is confirmed by the GO biological process “Negative regulation of chromosome organization” (Figure 7A).

#### 2.4.6. NLI Impairs Muscle Contraction via the Downregulation of Pathways Related to Calcium Signaling, Thyroid Hormone, and Circadian Clock

The investigation of the downregulated genes helped us to find the main reasons of the long-term cardiomyocyte pathologic remodeling and functional impairment. Glay clustering revealed a cluster containing 47 genes enriched in the gene module related to muscle contraction and several modules regulating this process, including “Regulation of Muscle system processes”, “Thyroid hormone synthesis”, “Circadian entrainment”, and “Calcium signaling pathway” (Figure 8A, Supplementary Figure S6, Supplementary Tables S2 and S7). The MCODE analysis identified the molecular complex related to calcium signaling and circadian clock regulated by Cacna1f, Cacna1d, Adcy1, Gng3, Plcb1, Rgs19 and other important genes (Figure 8B,C, Supplementary Figure S6, Supplementary Tables S2 and S7). The simultaneous downregulation of the gene modules related to muscle contraction and thyroid hormone biosynthesis is in a good agreement with the previously observed association between the hypothyroid state and the impairment of muscle contraction due to atrophy and fetalization of myofibrils [94]. In accordance with these data, our results revealed also the upregulation of JAK-STAT and TGF beta signaling pathways, (Figure 5 and Figure 6) which are known to promote cardiomyocyte fetal phenotype [77,95]. One more manifestation of the cardiomyocyte fetal phenotype is the shift of the expression ratio between the adult and fetal myosin heavy chain isoforms (Myh6 to Myh7) toward the fetal (Myh7) isoform [96]. From our results, this ratio is 3.19 folds in the control and 2.01 folds in the experiment. It is also important to note that the overexpression of Myh7, which is a reliable marker of cardiomyocyte atrophy and fetal gene program, is also known to accompany the hypothyroid state [36,96,97]. The disruptions of circadian clock and the downregulation of calcium signaling (Figure 8A–C) also can impair muscle contraction [98]. Recent studies indicate that these changes can be linked to the decrease in cardiac function and the pathogenesis of heart disease in response to adverse stresses and diseases [98,99,100]. Thus, our results indicate that NLI can trigger the long-term impairment of cardiac muscle contraction via well-established pathogenic mechanisms, including hypothyroidism, impairment of calcium signaling, and circadian clock disruption.

#### 2.4.7. Validation of mRNA-Seq Data of the Experimental vs. Control Expression Difference for Egr1, Tgfb2, and Ccna2 by qRT-PCR and Protein Interaction Analysis

The focus on the validation of genome-scale expression studies stems from prior work with microarrays [101]. Yet, RNA-seq does not suffer from the same issues as (some) microarrays did [101]. The microarrays technique is based on the hybridization of mRNA with a very short microarray probe. The RNA-seq uses the direct sequencing of several-fold longer mRNA sequences. The overall conclusion is that RNA-seq methods are robust enough to not always require validation by qPCR and/or other approaches [101]. Furthermore, we used the multi-step filtering of obtained switched on/off DEGs, based on the protein interaction network analysis, extracting the strongest effects and excluding possible spurious hits. However, we also did the direct validation of the main results using qRT-PCR and further protein interaction analysis.

To verify the association between the NLI and the long-term cardiomyocyte atrophy and fetal gene program, we validated the results of mRNA sequencing by qRT-PCR. To do this, we evaluated the NLI-related expression changes of the Tgfb2, Egr1, and Ccna2 genes. Tgfb2 is a marker of cardiomyocyte fetal phenotype, remodeling, and epithelial-to-mesenchymal transition [102,103]. Moreover, proteins of the TGFβ family can promote cardiomyocyte atrophy and synthesis of fetal contractile proteins, including slow myosin heavy chain Myh7 [103,104,105]. Egr1 is a marker of cardiomyocyte differentiation and hypertrophy that activates expression of the adult and fast myosin heavy chain isoform Myh6 [106,107,108]. Ccna2 regulates the cell cycle and promotes cardiomyocyte hypertrophy in the post-mitotic state [74]. Thus, we verified the expression of the fetal phenotype and atrophy-related marker Tgfb2 and the markers of cardiac hypertrophy Egr1 and Ccna2. The data of qRT-PCR indicated that the expression changes of all examined genes are in good agreement with the data of RNA-seq (Figure 9). The marker of atrophy and fetal gene program Tgfb2 is upregulated, whereas the markers of hypertrophy Egr1 and Ccna2 are downregulated, thus confirming the association between the NLI and long-term cardiomyocyte atrophy and fetalization. 

To further verify the expression difference between the experiment vs. the control for Egr1, Tgfb2, and Ccna2, and to characterize the biological context and interconnections of these markers with their partners, we constructed the protein interaction networks for the genes Egr1, Tgfb2, and Ccna2 with their twenty closest interactants using the String server [63]. Then, we defined and marked the experiment vs. the control expression difference for each protein in these three networks (Figure 10A–C). It can be seen that Egr1 decreases expression together with Egr3, Nab1, Nab2, and Srf (Figure 10A), which are known to be the direct regulators of Egr1 [109,110,111]. Figure 10B shows that Tgfb2 is upregulated together with Tgfb1,3 and Smad 3,4, which are known to be the key activators of Tgfb protein family [112]. At the same time, Smad6, which is an inhibitor of Tgfb proteins [112], shows strong downregulation. In accordance, Ccna2 demonstrates the downregulation (Figure 10C) together with the upregulation of its inhibitor Cdkn1a [74,113]. Thus, Figure 10A–C confirm that the data on the experiment vs. the control gene expression difference obtained with mRNA sequencing for Egr1, Tgfb2, and Ccna2 are in agreement with the changes of expression of their interactants. These observations confirm that Egr1, Tgfb, and Ccna2 perform gene regulation in coherence with their close partners and point to the reliability and accuracy of the mRNA sequencing.

#### 2.4.8. NLI Is Associated with Long-Term Deprivation of Glutathione Signaling, Detoxification, and Energy Metabolism

The results of gene module analysis indicated that NLI is associated with the long-term deprivation of glutathione metabolism and detoxification (Figure 11A–C, Supplementary Figure S7, Supplementary Tables S2 and S8). The MCODE analysis revealed the molecular complex related to glutathione signaling containing the well-known markers of glutathione metabolism (Gstm4, Gstm1, Gpx1, Gsta1, Gpx7, Gss, Gpx). These results point to the association between NLI and long-term heart pathology because the decreased activity of the gene modules related to glutathione metabolism, metabolism of lipids, and aerobic respiration are well-known features of cardiovascular diseases and hypertension [74,98,114]. Moreover, it was indicated that the decreased activity of antioxidant system affects the cell differentiation state and can change the cell fate [115,116,117]. In accordance, glutathione deficiency was associated with structural cardiac abnormalities in patients with heart diseases and patients with still undetected cardiovascular pathologies, thus suggesting that a blood test including glutathione measuring can be used as a biomarker for asymptomatic cardiovascular disease detection [118,119]. Thus, our results provide evidence that NLI can cause the long-term impairment of cardiac metabolism and antioxidant system.

## 3. Discussion

### 3.1. Significance of NLI Studying, Main Findings, and Research Status

The aim of this study was to verify the hypothesis suggesting that neonatal gastroenteritis caused by NLI plays an important role in the developmental programming of adult cardiovascular diseases. This hypothesis is based on the literature, indicating that, on the one hand, gastroenteritis can trigger the well-established factors of developmental programming of diseases, including inflammation, malnutrition, and growth retardation [10,14,18,20]. On the other hand, this hypothesis is based on the data indicating that lactose intolerance, which accompanies gastroenteritis of various etiologies, can cause inflammation, premature aging, growth retardation, and cachexia [20,44,45]. These effects originate from a causal link between lactose intolerance and oxidative stress, metabolic alterations, and extensive DNA damage caused by increased concentration of lactate and galactose in blood and intestine [42,44]. Despite the obvious danger of these effects to the developing heart, the consequences of NLI for cardiomyocyte maturation, polyploidization, and cell cycle exit, which altogether determine cardiac regenerative potential, remain unclear. Thus, the investigation of the long-term effects of NLI on maturating neonatal hearts will help to reveal new molecular and cellular mechanisms of cardiomyocyte cell cycle regulation and new triggers of developmental programming of adult cardiovascular diseases.

The bioinformatic analysis of mRNA-seq data indicated that NLI triggers the long-term gene expression changes that are known to accompany cardiac pathologies. Thus, the NLI manifestations found among the upregulated genes, including DNA instability, telomere damage, and inflammation are well-known markers of cardiomyopathies, heart failure, and hypertension [120,121,122,123]. DNA instability and telomere damage related to oxidative stress induce cardiac dysfunction and disease in experimental animals and in patients [74,124]. Moreover, the accumulation of unrepaired DNA breaks plays a causative role in the pathogenesis of heart failure. The molecular mechanism of this phenomenon includes the ability of persistent DNA damage to induce inflammation via cytokine overproduction [74]. Our data revealed that both DNA instability and features of inflammation accompany NLI, suggesting that NLI can cause long-term serious cardiac complications.

The NLI features identified among the downregulated genes, such as decreased activity of signaling via thyroid hormone, calcium, glutathione, and circadian clocks, are also well-known manifestations of heart diseases [121,122,123]. The hypothyroid state increases the risk of cardiac complications because it rapidly induces the mishandling of calcium signaling and contractile dysfunction, as well as cardiomyocyte atrophy and pathologic remodeling [94]. These pathologic changes occur because thyroid hormone deficiency downregulates fast (adult) myosin heavy chain α (Myh6), upregulates slow (fetal) myosin heavy chain β (Myh7), and downregulates sarcoplasmic/endoplasmic reticulum calcium ATPase 2 (Serca2a) and early growth response (Egr1), thus decreasing reuptake of calcium into the sarcoplasmic reticulum [125,126]. The deficiency of glutathione signaling originating, for example, from systemic inflammation, relates to heart failure progression and cardiac remodeling in animal models and patients with heart disease and heart failure [118,127]. Moreover, the decrease in glutathione blood level is the best diagnostic marker of subclinical heart diseases [118]. In accordance, the disruption of circadian-clock signaling results in abnormal cardiac metabolism, cardiomyocyte remodeling, and dilated cardiomyopathy [128]. Taken together, the identified transcriptomic changes present a comprehensive picture of the pathological features associated with NLI, because they are all interconnected by causal relationships. Thus, systemic inflammation decreases glutathione-dependent oxidative stress defense, causes massive DNA instability, and impairs thyroid hormone signaling, thereby disrupting cardiomyocyte contraction.

Beyond the association between NLI and the well-established features of heart pathology in the main signaling pathways, our data also revealed the morphologic and transcriptomic traits of increased polyploidy that are also known to accompany various heart diseases, including tetralogy of Fallot, cardiomyopathy, hypertension, and ischemic heart disease [14,51,89,129,130,131]. Among these features are the response to DNA and telomere damage and the impairment of circadian entrainment [73,132,133,134]. Our data also uncovered ploidy-related manifestations of the fetal phenotype in the signaling pathways and metabolism [88,135,136,137]. For example, the associations between polyploidy and the activation of pathways of meiosis, female gametogenesis, programs of unicellularity, and signaling of multipotency (TGFbeta, JAK-STAT, c-KIT) were well documented in heart diseases, cancer, and normal tissues [79,89,134,138,139,140,141,142,143,144]. Our data also identified the traits of open chromatin, such as the upregulation of the gene module “Negative regulation of chromosome organization” and modules related to active transcription and ribosome biogenesis. The association between polyploidy, chromatin opening, ribosome biogenesis, and active transcription was previously described in several studies performed with mammalian and plant cells in normal and pathologic states [91,145,146,147].

Importantly, we showed these NLI-induced effects in the most strongly deregulated genes (switched on/off). Such discrete changes may suggest that the genome is undergoing substantial alteration possibly related to chromatin remodeling [52,53,54]. The on/off gene switching is more typical for development and cancer cells, which experience profound changes [53,54,55,56]. The on/off switch in the NLI response suggests that it involves qualitative alteration in gene expression.

The ability of polyploidy to open chromatin reactivates the fetal gene programs, thereby increasing cell biological plasticity via activation of bivalent genes, enabling cells to fast regulatory network rewiring in response to stress or environmental clue [46,88,91,145]. On the one hand, this ability is beneficial, because it helps to cope with extreme stress, including DNA damage, oxidative stress, hypoxia, inflammation, and others; on the other hand, it is detrimental because it impairs cell specific functions, including cardiac contraction [89,134,148,149]. Extensive comparative and experimental studies confirm that polyploidy impairs cardiac functions, even despite its ability to increase stress resistance [139,150,151]. Because cardiomyocyte polyploidization is irreversible [51], the detrimental effects of hyperpolyploidy in lactose-fed animals may have long-term, or even lifelong detrimental effects for cardiac performance. Therefore, we can consider cardiomyocyte hyperpolyploidization as an important epigenetic mechanism of developmental programming of adult cardiovascular diseases, and lactose intolerance can be related to the important factors triggering this and other mechanisms of developmental programming. Thus, our data can contribute to preventive medicine considered as the medicine of the future [152,153,154,155].

### 3.2. Strength, Possible Limitations, Future Directions, and Medical Applications of the Study

In this study, we provided first evidence that NLI can be an important mechanism of developmental programming of adult cardiovascular disease. We identified the molecular nature of this interconnection by indicating that NLI operates via polyploidy and DNA instability. We also provided a comprehensive and cohesive picture of long-term transcriptome-wide traits associated with neonatal lactose intolerance (NLI). Our new bioinformatic approach focused on the analysis of gene modules, including hub genes and molecular complexes, enabled us to identify both causes and consequences of NLI. Thus, NLI-associated DNA instability, cardiomyocyte atrophy, inflammation, fetal gene program, and fibrosis may originate from the impairment of antioxidant system, hypothyroid state, and calcium signaling.

There are also possible limitations to this study. Here we performed an investigation with the neonatal rat model. Small rodents differ from humans by degree of maturity at birth and cardiac growth trajectory [24,156,157,158]. Therefore, although lactose intolerance is worldwide in human babies, infants, and children, we still do not know to what extent our results can be translated to humans. In addition, we investigated here cardiomyocytes from the LV only, whereas the response of cardiomyocytes from other chambers remains unknown. The spatiotemporal developmental programs of cardiomyocyte maturation differ between atria and ventricle [23]. Because atria and right ventricle undergo terminal differentiation later in postnatal ontogenesis than LV [159,160], it is quite possible that after the investigation of atria and right ventricle, the critical developmental window identified here may be extended.

The future perspective of the study includes the experiments with larger animals aimed at the investigation of the short-term and long-term effects of NLI on cardiac anatomy, function, transcriptome, and epigenome. The focus on the long-term effects of NLI on the pathways related to cardiomyocyte maturation, stress response, and senescence will illuminate factors driving cardiac postnatal organogenesis and regeneration. It would be also exciting to find out how various concentrations of lactose affect iPSC-derived cardiomyocyte maturation in culture.

It is also important to note that, although cardiovascular diseases are a leading cause of death and disability worldwide, the roles for neonatal inflammatory stress in long-term cardiovascular health are still far from complete understanding. The results of this study identified a new important trigger of early life systemic inflammation that can increase the risk of cardiovascular diseases later in life. The clinical application of the presented results will help in the identification of potential strategies, which can be used to mitigate the negative effects of NLI for cardiac health.

### 3.3. Conclusions

In conclusion, here we presented a novel rat model of NLI characterized by animal growth retardation and cardiomyocyte hyperpolyploidy and atrophy. Extensive bioinformatic analysis revealed the association between NLI and well-known features of heart pathology, including DNA and telomere instability, inflammation, manifestations of fetal gene program, fibrosis, and impairment signaling via thyroid hormone, calcium, and glutathione. We also found transcriptomic manifestations of increased cardiomyocyte polyploidy, such as the induction of gene modules pointing to chromatin relaxation. These modules include “negative regulation of chromosome organization”, “transcription”, and “ribosome biogenesis”. The long-term induction of these gene modules suggests that ploidy-related epigenetic changes acquired in the neonatal period permanently rewire gene regulatory networks and alter cardiomyocyte transcriptome. Our NLI model may be useful for disentangling the complex relationship between neonatal systemic inflammation, disturbed cardiac organogenesis, and the long-term increased risk of cardiovascular diseases.

## 4. Materials and Methods

### 4.1. Animals

The adult rat females and males weighting 180–250 g were purchased from the Rappolovo nursery of the Russian Academy of Medical Sciences. To obtain newborn pups, female rats were bred overnight with age-matched males. At the day of birth, the pups from several dams were sorted to standardize litters to eight animals (four males and four females). Control pups were housed in an environmentally controlled facility on a 12:12 h light:dark cycle and were fed a standard laboratory diet and water ad libitum as recommended in [161].

### 4.2. Statement of Ethics

The study was conducted according to the guidelines of the Declaration of Helsinki. All animal procedures were carried out in accordance with the Animal Welfare Assurance (Assurance Identification number F18-00380) of the Institute of Cytology, Russian Academy of Sciences for the protection of animals that are reared at experimental farms and used for scientific purposes. All experimental protocols were also in compliance with the US Department of Health and Human Services Guide for the Care and Use of Laboratory Animals (1996) [162].

### 4.3. Experimental Design and Lactose-Containing Diet

To induce lactose intolerance in suckling rat puppies, we overloaded intestinal enterocyte lactase capacity. To do this, 8-day-old animals were fed with lactose-containing water until weaning at day 21. This particular time window was chosen because it coincides with the critical period of neonatal cardiomyocyte maturation when cells switch from proliferation to polyploidization [14,139]. Lactose was purchased from Vecton (St. Petersburg, Russia). The daily lactose amount per rat puppy was calculated according to the data on age-specific lactase safety factor and age-specific amount of lactose consumption with milk. The lactase safety factor is determined by the ability of the enzyme to hydrolyze lactose into monosaccharides glucose and galactose and by the capacity of Na^+^-glucose cotransporter (SGLT-1) of the intestinal brush border to transport monosaccharides across the brush border membrane. In the rats aged 9–15 and 16–21 days after birth, the lactase safety factor is about 2 folds and 1 fold, respectively. The daily lactose consumption with milk is about 0.5 and 0.7 mmol, respectively [161]. Thus, to overload lactase capacity, the animals aged 8–16 and 16–20 days weighting 27–40 g and 43–54 g received 2 doses and 1 dose of usual daily lactose consumption equal to 2 × 0.5 mmol (342 mg) and 1 × 0.9 mmol (307 mg), respectively. To successfully feed the animals, lactose was dissolved in 1.5 mL of distilled water. Then, lactose-containing water was heated up to 37 °C and 0.25 mL (or approximately 5 drops) of water was slowly administered by oral plastic flexible gavages with intervals of 1 h during 6 h (from 10 am to 6 pm). The liquid reminded milk because of incomplete lactose dissolution. Every time of feeding, the experimental animals willingly approached the hands of a laboratory assistant and ate lactose with pleasure, which shows the comfort of the experimental conditions. Control animals received the same amount of distilled heated water in parallel. The investigation of postponed effects of neonatal lactose feeding was performed in 140-day-old animals, i.e., 4 months after treatment. All animals were weighed at day 140 after birth. Overall, 24 control and 24 experimental animals were investigated.

### 4.4. Cardiomyocyte Isolation

To obtain isolated cardiomyocytes, rats were sacrificed by intraperitoneal injection of ketamine (100 mg/kg) and xylazine (10 mg/kg). Then, the hearts were removed. Isolated cardiomyocytes were prepared by retrograde coronary perfusion using enzymatic protocol with collagenase from [163], with small modification. For this propose, the whole hearts were perfused with Tyrode solution (buffer1) in mM: (140 NaCl, 6 KCl, 10 glucose, 10 HEPES, 1 MgCl_2_, 1 CaCl_2_; pH 7.4), for 3 min, and then the hearts were perfused with low Ca2^+^ solution (buffer 2) for 5 min. In mM: (120 NaCl, 5.4 KCl, 5 MgSO_4_, 5 sodium pyruvate, 20 glucose, 20 taurine, 10 HEPES, 5 nitrilotriacetic acid, and 0.04 CaCl_2_; pH 6.96) for 5 min, and for 9 min with solution containing collagense II (1.1 mg/mL; Sigma-Aldrich, St. Louis, MO, USA) and hyaluronidase (0.5 mg/mL; Sigma-Aldrich) solved in buffer 1 and dissected to separate the left ventricle from the right ventricle and atria. Then, a small tissue fragment of LV apical part was cut into small pieces and resuspended gently to release cells. After gravity sedimentation, cells were placed in a buffer containing 10% calf serum and 12.5 μM CaCl_2_ to stop further digestion, as recommended in [164] (Auguste, et al., 2018). Then the cells were filtered through a 200 μm nylon mesh to remove undigested tissue fragments. Collected cells were allowed to sediment by gravity. The concentration of cells in suspension and the degree of their damage were tested under phase-contrast microscope. After that, 5 drops of suspension were placed on a glass slide and cell smears were prepared. After air drying, the preparations were fixed with absolute methanol.

### 4.5. Cardiomyocyte Staining and Ploidy Evaluation

Cardiomyocyte ploidy was evaluated after staining of cell smears with 20 µg/mL Hoechst 33258 water solutions for 15 min. For this purpose, 3–5 drops of the staining solution were dipped on the smears and covered with cover slips. The quantitative image analysis was conducted with a Zeiss Axioskop microscope equipped with a digital CCD video camera VarioCam (PCO Computer Optics GMBH, PCO-Tech, Kelheim, Germany), using Image J program package. At least 500 cardiomyocytes were evaluated for 24 experimental and 24 control rats. Cardiomyocyte ploidy levels were determined using splenocytes of the same animals as reference cells with diploid DNA quantity. The proportion of cardiomyocytes in S-phase did not exceed 1.5% in postnatal rats [165]. Therefore, in order to distinguish the S-phase cells from the polyploid ones, we excluded cells with ploidy not divisible by 2n (±10%). Average cardiomyocyte genome number was calculated by the formula taken form [14,150].

PLD = ∑i × n_i_

where PLD is the mean number of genomes per cell, n_i_—the fraction of cardiomyocytes of the i-class of ploidy.

### 4.6. Cardiomyocyte Morphological Features of DNA Instability

Cardiomyocyte DNA instability was identified by bridges between nuclei [57,58] and by the percentage of aneuploid cells, i.e., cells with aneuploid nuclei containing abnormal number of genomes [59]. These features were assessed in isolated cells using a Zeiss Axioskop microscope with a digital CCD video camera VarioCam and Image J program package. In all, 500 cells were evaluated from 24 experimental and 24 control animals. 

### 4.7. Cardiomyocyte Protein Content Evaluation

To evaluate protein content in isolated cardiomyocytes, cell smears were stained with 0.1% naphthol yellow in 1% acetic acid for 30 min and washed in three changes of 1% acetic acid and in three changes of 100% isobutanol for 3s at room temperature [14]. The protein content was evaluated on the cardiomyocyte images obtained with an Axio Scope microscope (Carl Zeiss, Jena, Germany) and Image J 1.40g software (National Institute of Health, Bethesda, MD, USA). For each animal, no less than 300 cells were evaluated. Each cell was processed three times by the “Mean Grey Value” parameter.

### 4.8. mRNA Sequencing

The mRNA sequencing was performed by Genotek Co, Moscow, Russia. For this purpose, total RNA was isolated from fragments of the rat cardiac left ventricle apical parts (weighting 10–15 mg) and stored at −80. RNA was extracted using PureLink RNA Mini Kit (AMBION, Life Technologies, Carlsbad, CA, USA). After that, mRNA was extracted from total RNA using magnetic beads (Sileks). cDNA libraries were prepared using NEBNext^®^ mRNA Library Prep Reagent Set for Illumina (New England Biolabs, Ipswich, MA, USA). In this approach, mRNA was fragmented, and cDNA was synthesized, end-repaired, and ligated to unique sequencing adapters to form cDNA libraries. The indexing was performed by PCR with NEBNext Multiplex Oligos for Illumina (dual index primers set 1). Quality control of prepared libraries was made using Bioanalyzer 2100 (Agilent Technologies, Santa Clara, CA, USA). Sequencing of cDNA libraries was conducted on HiSeq2500 (Illumina, San Diego, CA, USA) in rapid run mode with read length 100 nt.

Next-generation sequencing was conducted by parallel measurement of three biological samples both for the control and lactose-treated animals. The NGS reads were trimmed using the “trimmomatic” software specially developed for Illumina NGS data, with default parameters [166]. The trimmed reads were mapped to canonical nonredundant rat transcriptome presented in the RefSeq database [167], using Bowtie 2 software, version 2.5.0 [168,169]. This aligner is a standard within mapping pipelines and shows a remarkable tolerance both to sequencing errors and indels [170]. Bowtie 2 was used with the “very sensitive” preset of parameters, which allows the most sensitive and accurate mapping (at the expense of speed). Only the non-ambiguous mappings were counted. The obtained counts were analyzed using the “limma” package (implemented in R environment) specially developed for whole-transcriptome analyses of differentially expressed genes [171]. The data normalization methods presented in limma (quantile, scale) were tested as well as the trimmed mean method from the edgeR package [172]. The results were similar. The results obtained with quantile normalization are presented here.

### 4.9. Validation of mRNA-Seq by qRT-PCR

To validate the data obtained by mRNA sequencing, we evaluated the expression difference for three genes (TGFB2, EGR1, and CCNA2) in the experiment vs. the control by qRT-PCR. To do this, we used left ventricle apical fragments (weighing 10–15 mg) from 5 control and 5 experimental animals. The tissue fragments were stored at −80 °C prior to RNA extraction. Total RNA was isolated using Trizol reagent (Invitrogene, Waltham, MA, USA) according to the manufacturer’s instructions. The quality of isolated RNA was estimated with electrophoresis in 1.5% agarose gel containing 5 μg/mL of ethidium bromide in TAE—buffer [173]. The purity and preservation of RNA were evaluated by the 18S and 28S ribosomal RNA bands sharpness observed in ultraviolet light. The amount of isolated RNA was determined spectrophotometrically by absorption of ultraviolet light with a wavelength of 260 nm [173]. The RNA preparation was purified from DNA by DNAse free RNase treating. Each reaction mixture of 40 μL contained about 10 μg RNA and 1 U DNase (DNase RNase—free; Roche—Boehringer—Mannheim, France). The reaction was carried out at room temperature for 30 min. The reaction mixture was deproteinized with phenol−chloroform mixture, and RNA was sedimented with ethanol [173]. To obtain complementary DNA (cDNA), RNA reverse transcription was performed with a set of reagents for synthesis of the first cDNA chain Revert AidTM (Fermentas, Lithuania) according to manufacturer instructions and within 24 h of RNA isolation. The quality of synthesized cDNA was checked by the PCR method. Samples were stored at a temperature of −80 °C.

The primers were constructed with the use of Primer 3.0 and Primer design software (NCBI, http://primer3.sourceforge.net/releases.php Version 2.4.0, assessed on 16 May 2022) or were taken from the literature. Synthesis of the primers was conducted by Sintol Co (the sequences of the primers are indicated in the Table 1). Quantitative estimation of mRNA transcription was performed by the ABI PRISMF 7700 Sequence Detection System (Applied Biosystems, Foster City, CA, USA). The obtained calibration curves appeared to be almost parallel for all genes, which confirms the approximately equal PCR efficiency and allows using ΔCt method [174] for the expression difference estimation. The reaction mixture (25 μL) contained 0.5 μL of the direct primer, 0.5 μL of the reverse primer, 1 μL of the cDNA matrix, and 10 μL of the tenfold stain Power SYBER Green (Applied Biosystems, Waltham, MA, USA). Each reaction was repeated three times at the following parameters: 5 min at 95 °C (for DNA−polymerase activation) and 50 cycles at 95 °C for 15 s, 60 °C for 15 s, and 72 °C for 50 s. The gene expression levels of Tgf-β2, Egr1 and Ccna were normalized by the expression levels of reference (housekeeping) gene Gapdh, using the ΔCt method [174].

### 4.10. Data Purification from Noise and Gene Module Analysis

To reveal the qualitatively deregulated genes, we selected the switched on/off genes (i.e., those that were expressed in experiment and were not expressed in the control, and vice versa).

The problem with determining whether the genes are switched on/off is that many genes can show expression not at zero level, but very close to it. However, it is not clear whether this expression is functional or just a noise. Therefore, we considered the peaks of gene density on a two-dimensional histogram as a switched on/off criterion (Figure 3). The gaps between the peaks show the qualitative (discrete) changes in expression corresponding to transitions between different states, i.e., switching the genes on/off.

To identify the biological pathways enriching DEGs with maximum stringency, we applied the further multi-step purification of signal from noise for switched on/off genes, which was based on the protein–protein interaction network analysis. This was conducted because we wanted to exclude possible spurious hits and to reveal the most important (and the strongest) effects in the experiment vs. the control. First, using the String server [63], we obtained protein–protein interactions of high confidence for switched on/off DEGs. This step gave 778 upregulated and 552 downregulated DEGs. Then, to reveal the most important genes (hubs), we selected nodes with a degree above 2 (i.e., nodes with >2 interactions). This step provided 496 upregulated and 355 downregulated genes. Then, to identify the tight functional gene clusters among these genes, we applied the “Glay clustering” algorithm implemented in the Cytoscape [61] (version 3.9.1), as previously [65]. This is a method of choice for our purpose because the Glay algorithm provides a complete clustering of large interaction networks [65,66,67]. The Glay clustering revealed the four large (containing above 10 genes) tight clusters among the switched on genes and the two such clusters among the switched off genes (Supplementary Tables S1 and S2). These clusters were analyzed with the Metascape server [62]. This server allows revealing the biological features by means of term, pathway, and process enrichment analysis. Moreover, Metascape performs protein–protein interaction (PPI) enrichment analysis with physical interactions and constructs the network containing the subset of proteins that form physical interactions with at least one other member in the list. Metascape identifies molecular complexes via the Molecular Complex Detection (MCODE) algorithm [69]. The Metascape analysis of the Glay clusters revealed alterations in the basic biological processes and functions in the experiment vs. the control (Figure 4, Figure 5, Figure 6, Figure 7 and Figure 8 and Figure 11). Thus, we supported the analysis of the switched on/off DEGs by the interactome analysis, revealing not just separate DEGs but DEGs forming protein interaction clusters. This procedure excludes possible spurious hits that may be associated with separate genes.

### 4.11. Tgfb2, Egr1, and Ccna2 Close-Interactants Network Construction

To confirm the results of the RT-PCR validation of the data obtained with mRNA-seq, we constructed protein–protein interaction networks for the validated genes Tgfb2, Egr1, and Ccna2 using the String server [63]. All three networks were constructed for the 20 closest interactants. Then we determined the experiment vs. the control expression difference for the genes encoding proteins from the networks, using the data of mRNA-seq and analyzed the functional meaning of the obtained results with particular focus to the direct regulators, inhibitors, or activators of Tgfb2, Egr1, and Ccna2.

### 4.12. Statistical Analyses

The statistical analyses were conducted using the GraphPad Prism 8, and Statgraphics Centurion 18, and R packages, as well as the Metascape server. For all figures, the statistical significance of differences between the experiment and the control is shown either directly in the figure or in the figure legend.

## Figures and Tables

**Table 1:** Sequence of primers.

Gene	Sequence
Glyceraldehyde triphosphate Dehydrogenase, (Gapdh); Gene ID 24383	Forward: 5′-GGGGGCTCTCTGCTCCTCCC-3′ Reverse: 5′-CAGGCGTCCGATACGGCCAA-3′
Early growth response, (Egr1); Gene ID 24383	Forward: 5′-CACCAGACCATGCTTCAGTGAGA-3′ Reverse: 5′-GTTGCATGGCTGTTCACAGGA-3′
Transforming growth factor β2, (Tgfβ2), Gene ID 81809	Forward: 5′-CTCCACATATGCCAGTGGTG-3′ Reverse: 5′-CTAAAGCAATAGGCGGCATC-3′
Cyclin A2, (Ccna2); Gene ID: 114494	Forward: 5′-ATGTCACCGTTCCTCCTTG-3′ Reverse: 5′-GGGCATCTTCACGCTCTATT-3′

## Data Availability

Raw data may be provided by reasonable request.

## Supplementary Materials

The following supporting information can be downloaded at: https://www.mdpi.com/article/10.3390/ijms24087063/s1.
