# Supplementary material for: Long-Term Transcriptomic Changes and Cardiomyocyte Hyperpolyploidy after Lactose Intolerance in Neonatal Rats
Source: Int J Mol Sci. 2023 Apr 11;24(8):7063. doi: 10.3390/ijms24087063 (PMC10138443; doi:10.3390/ijms24087063)
Supplement: Supplementary file 1 [file ijms-24-07063-s001.zip › Supplementary Figures S1-S7.docx]

**Supplementary Figures S1–S7**

**
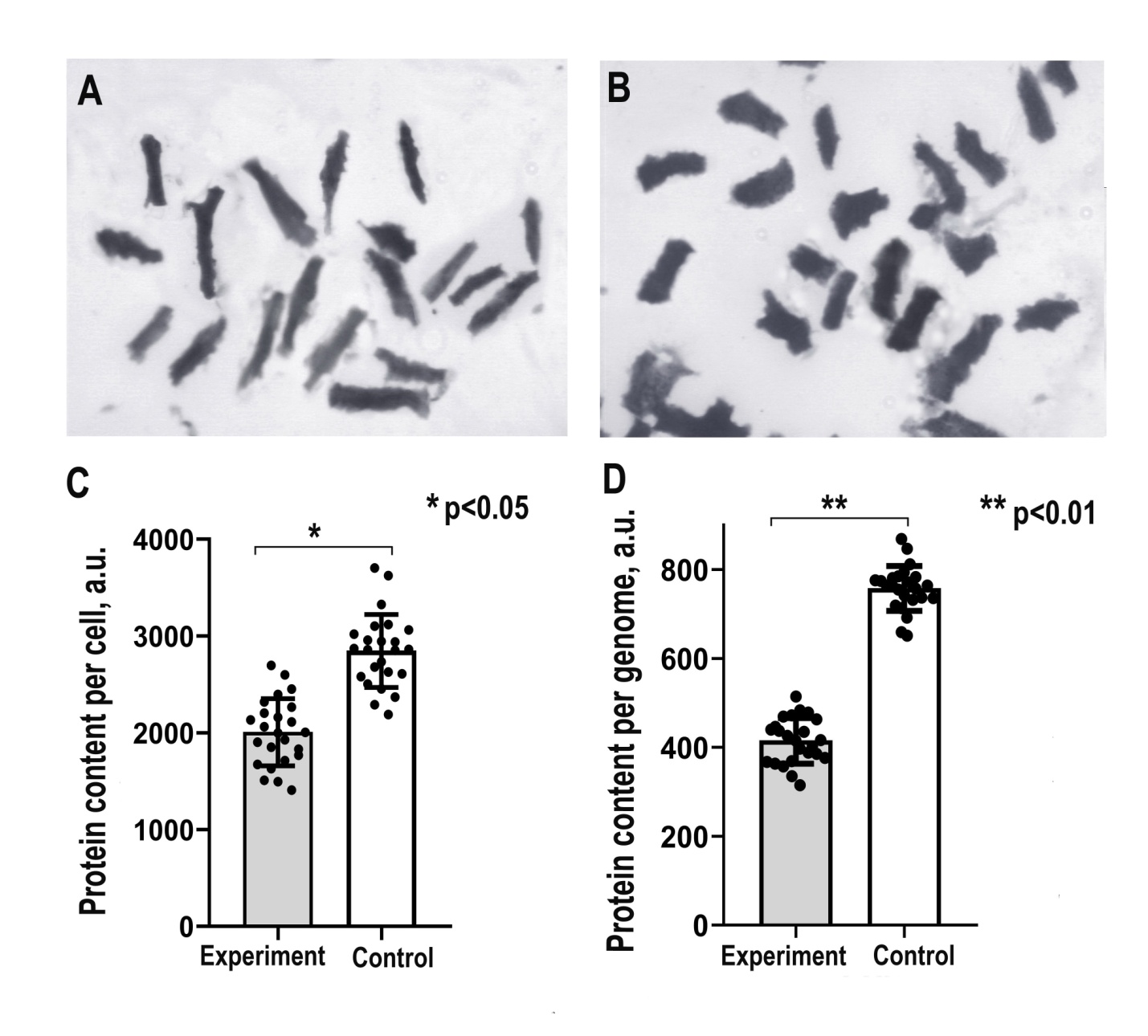
**

**Supplementary Figure S1**. Cardiomyocyte protein content in the experiment and in control. (**A**)–cardiomyocytes from the experimental animal; (**B**)–cardiomyocytes from control. Staining with naphthol-yellow, total magnification x100. (**C**)–Cardiomyocyte protein content per cell. (**D**)–Protein content per genome of cardiomyocyte. The bars represent mean values; the error bars show confidence intervals (CI); points represent separate values.

**
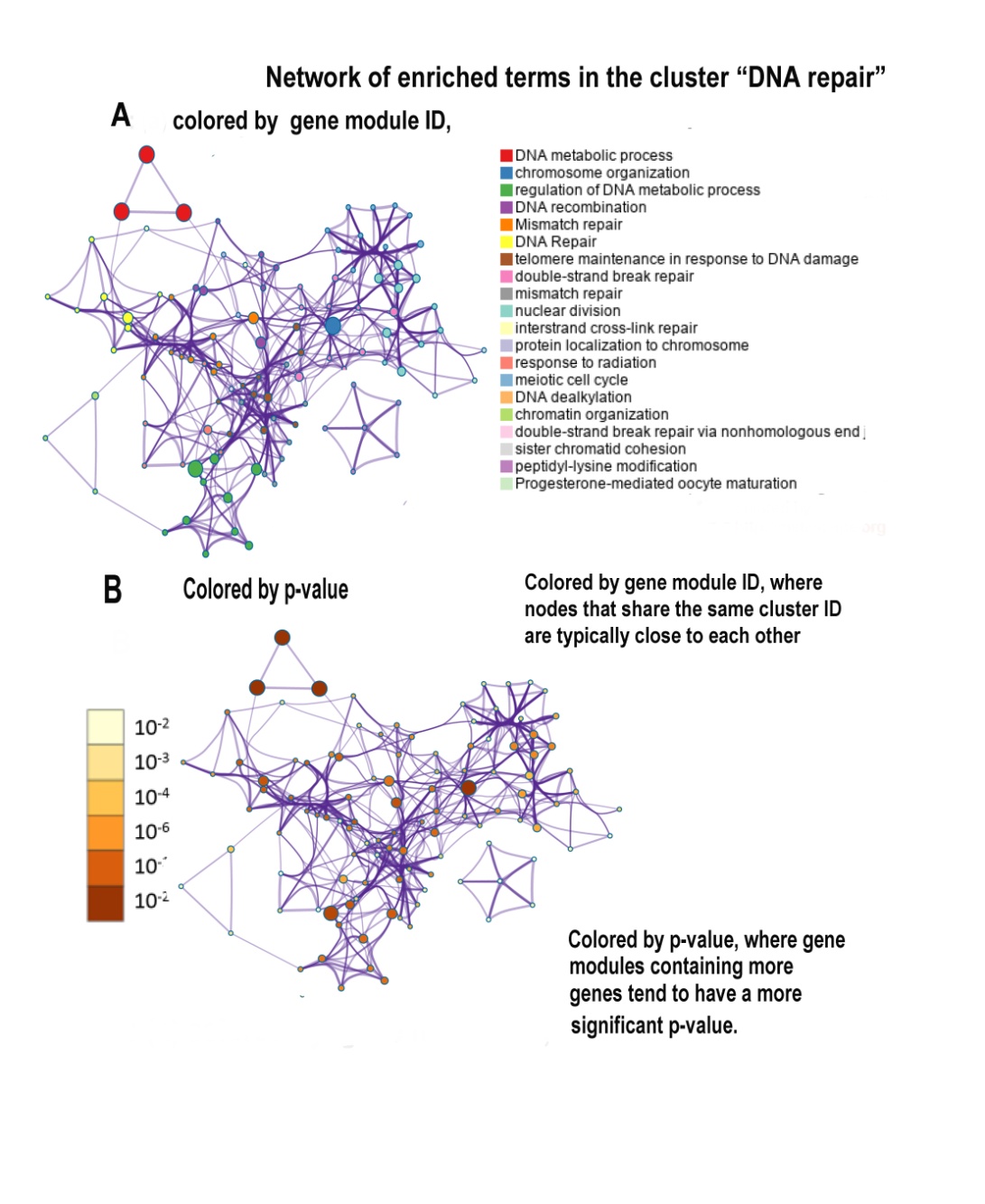
**

**Supplementary Figure S2**

**
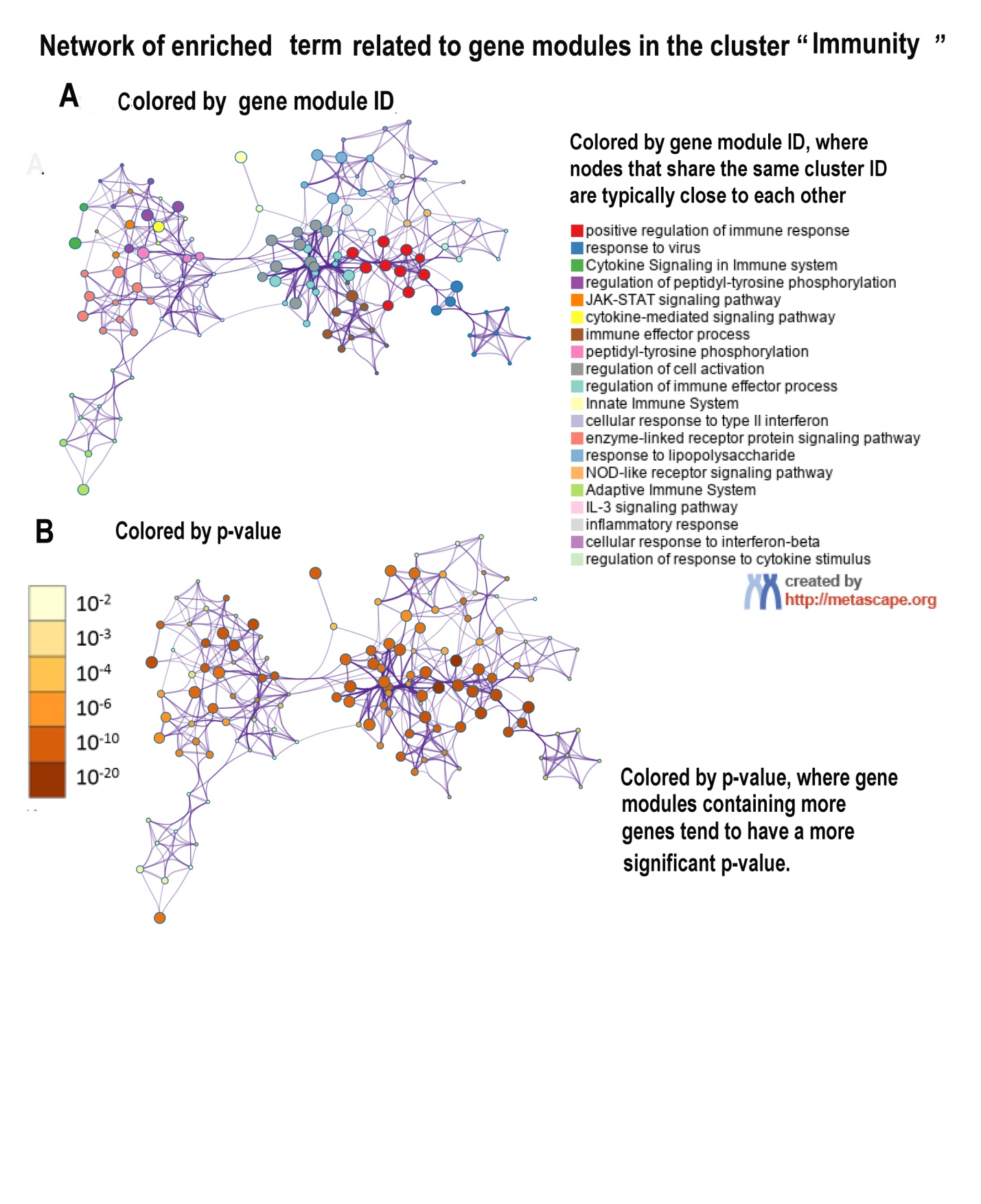
**

**Supplementary Figure S3**

**
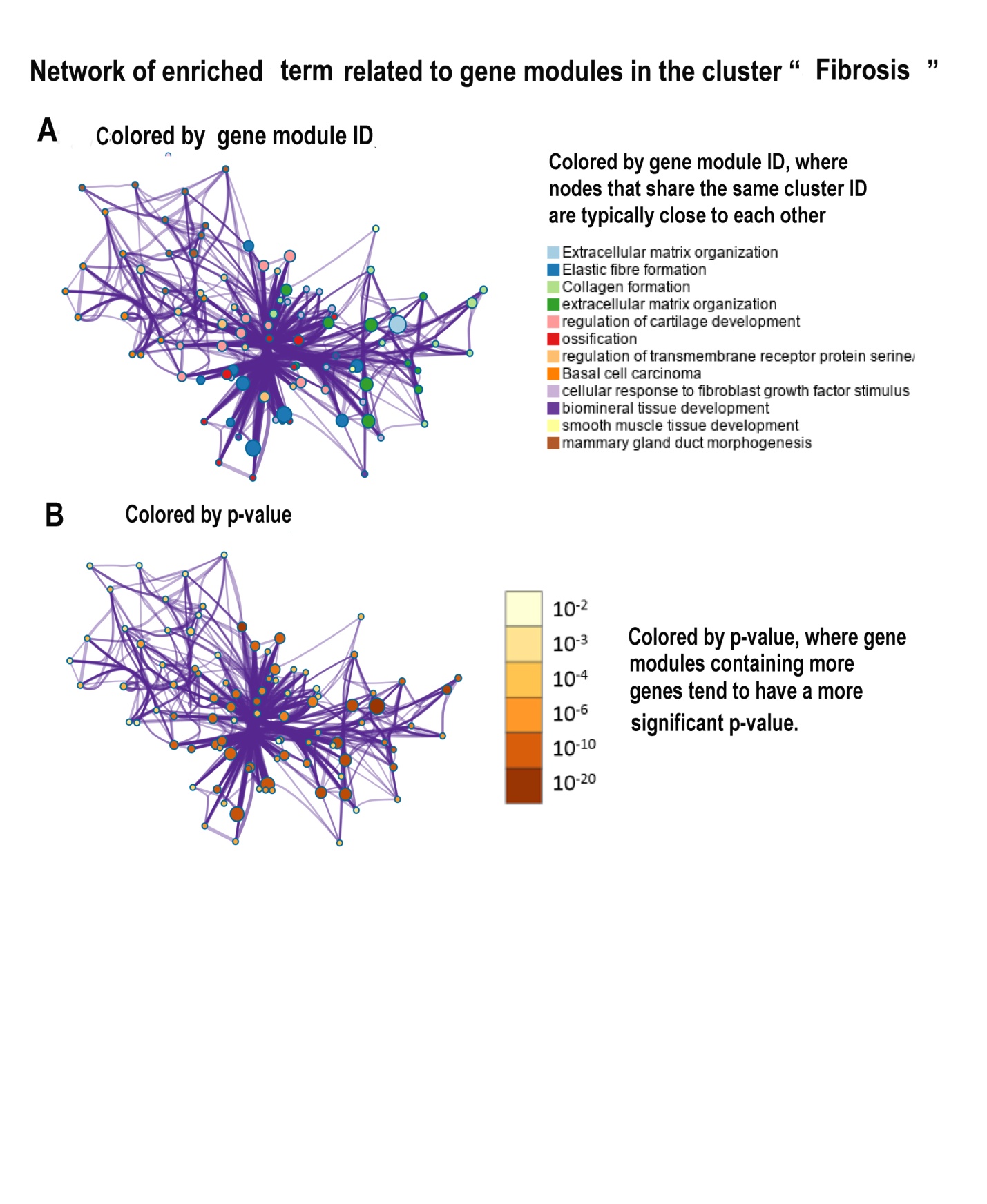
**

**Supplementary Figure S4**

**
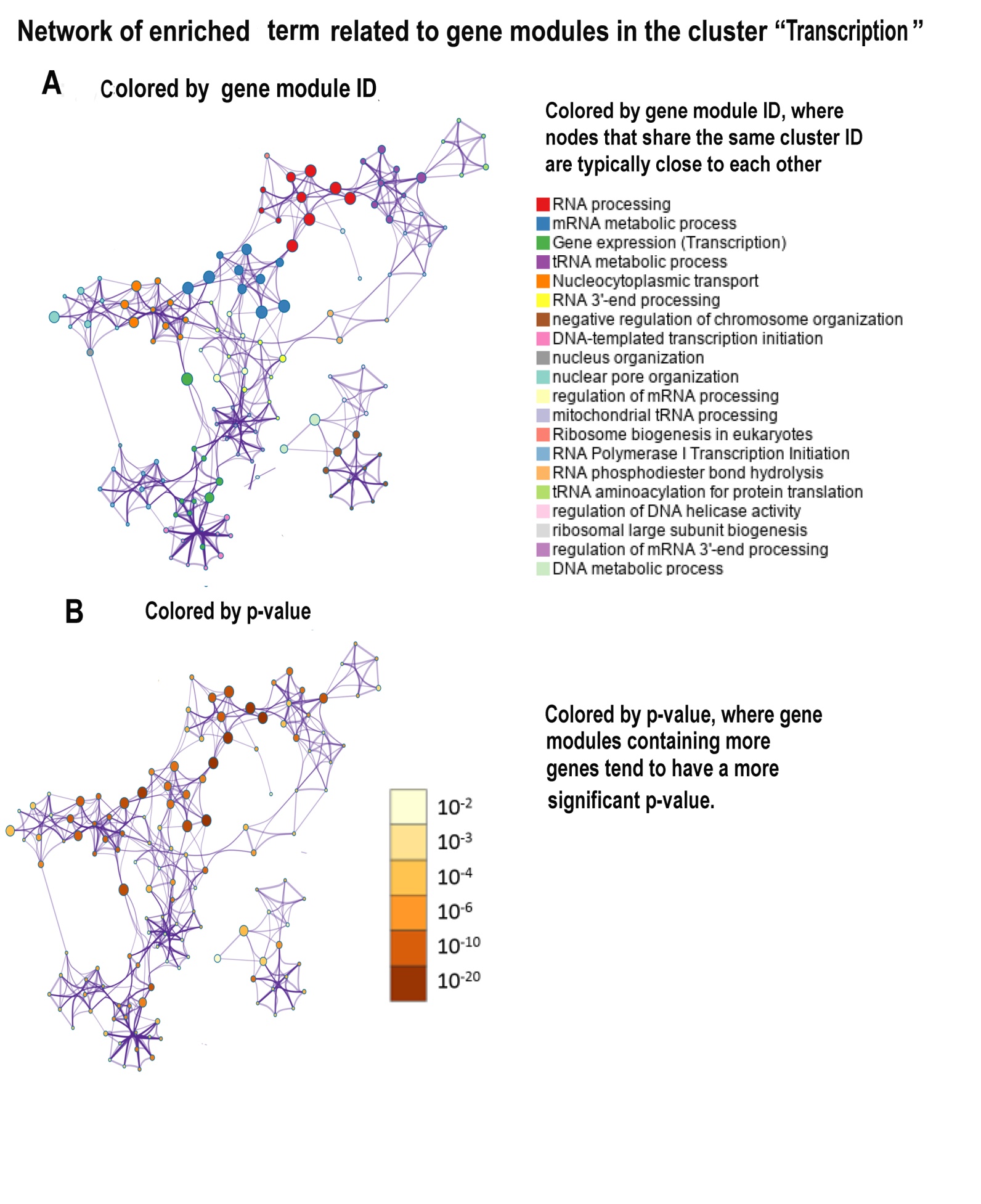
**

**Supplementary Figure S5**

**
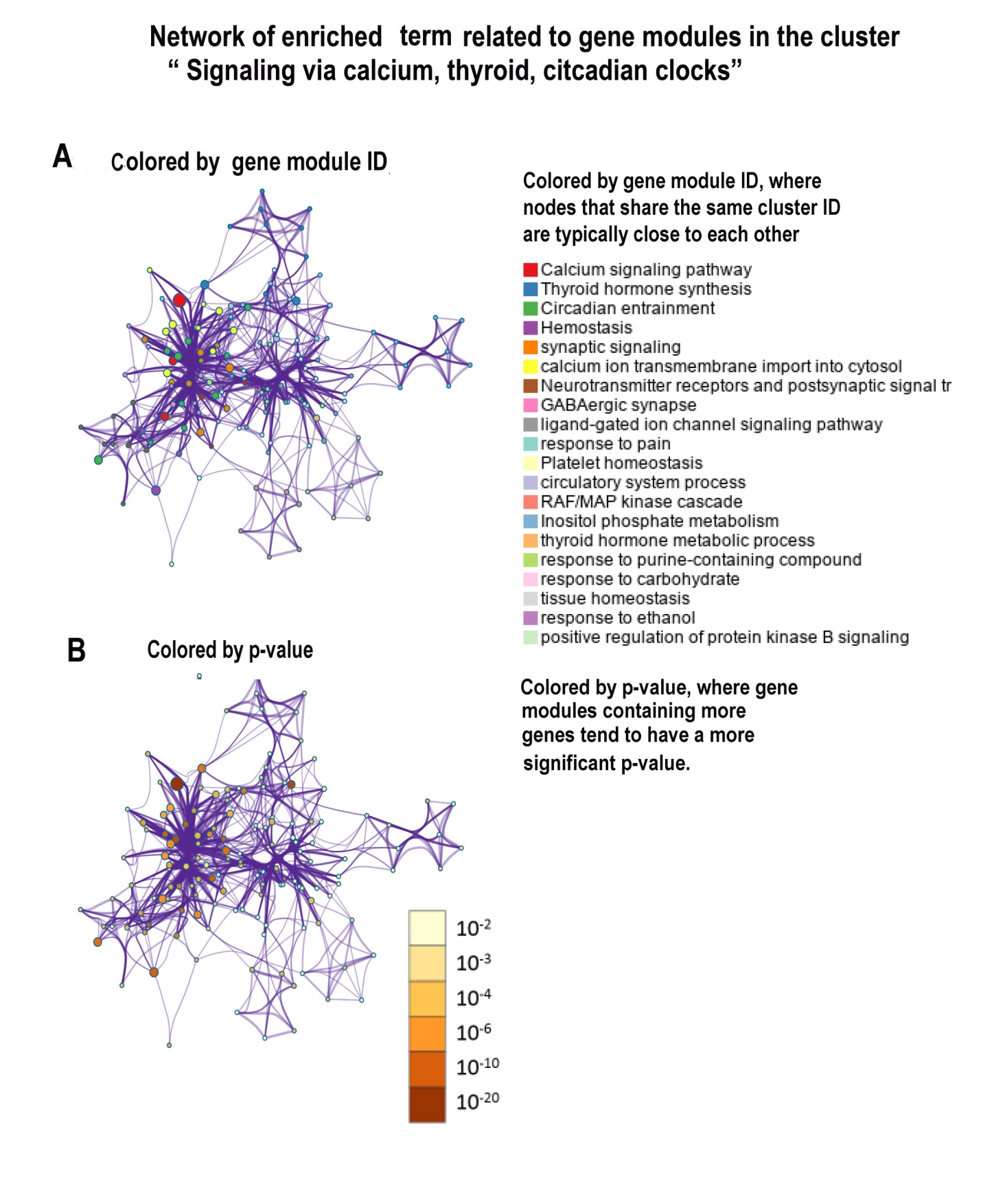
**

**Supplementary Figure S6**

**
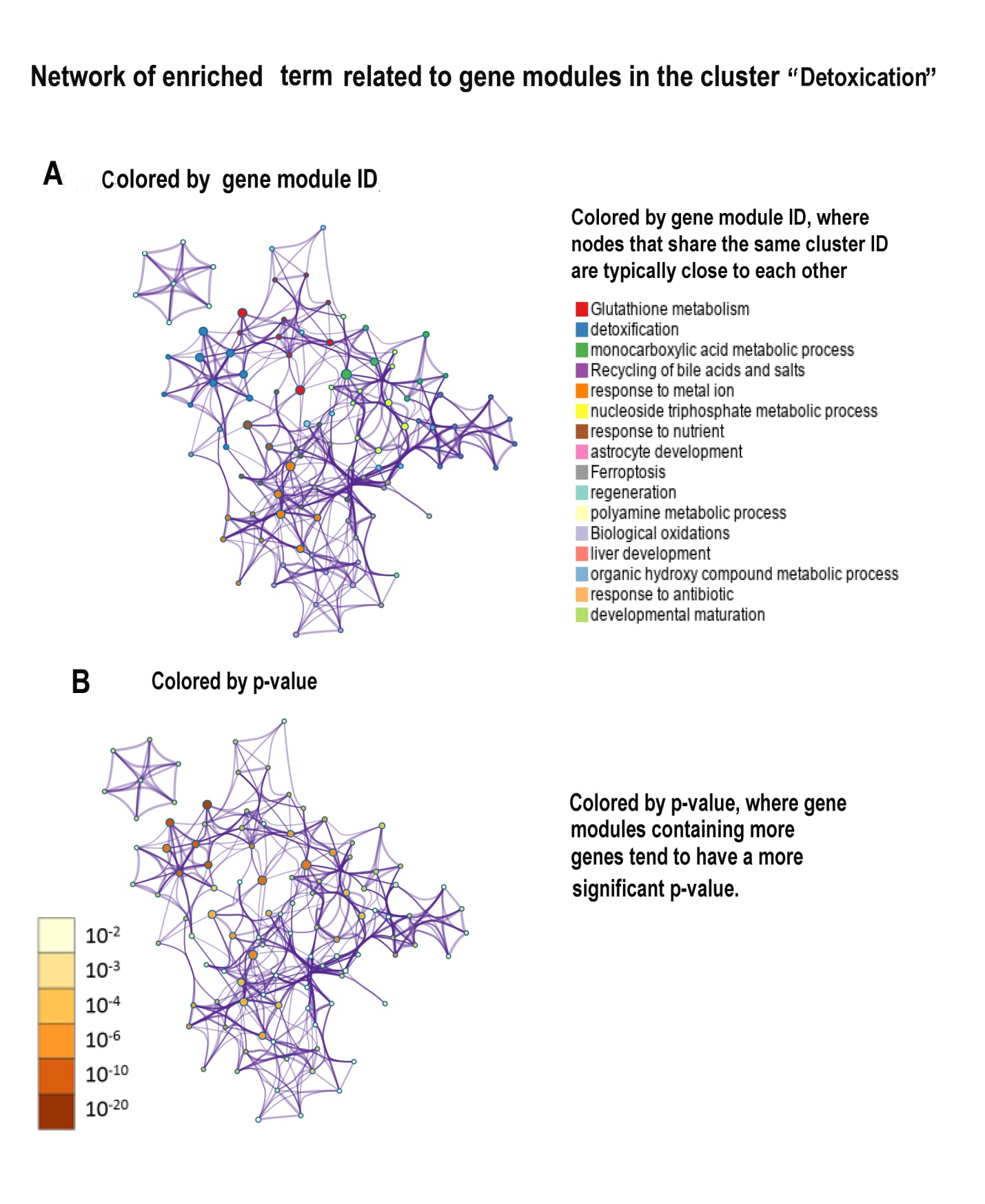
**

**Supplementary Figure S7**
